# Supplementary material for: Overcoming primary and acquired resistance to anti-PD-L1 therapy by induction and activation of tumor-residing cDC1s
Source: Nat Commun. 2020 Oct 27;11:5415. doi: 10.1038/s41467-020-19192-z (PMC7592056; doi:10.1038/s41467-020-19192-z)
Supplement: Supplementary file 1 — Supplementary Information [file 41467_2020_19192_MOESM1_ESM.pdf]

Overcoming primary and acquired resistance to anti-PD-L1 therapy by induction and activation of tumor-residing cDC1s

Oba *et al*.

Supplementary Information

Supplementary Figures 1-21
Supplementary Tables 1-3

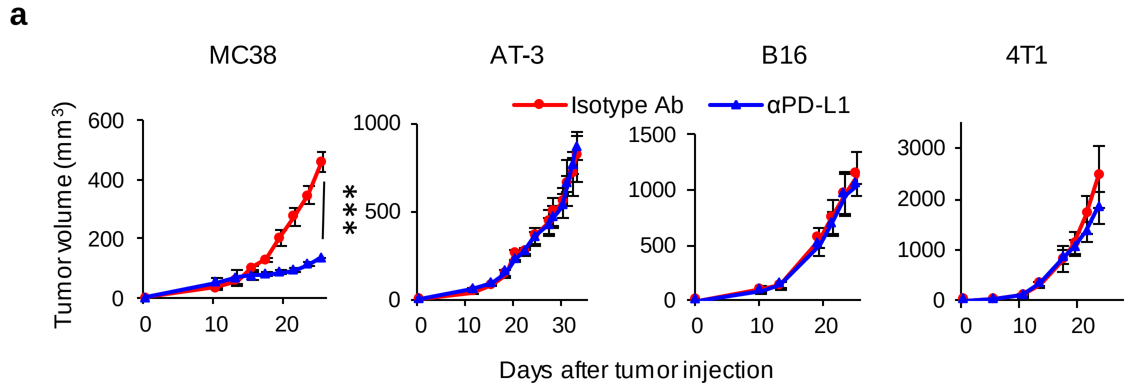

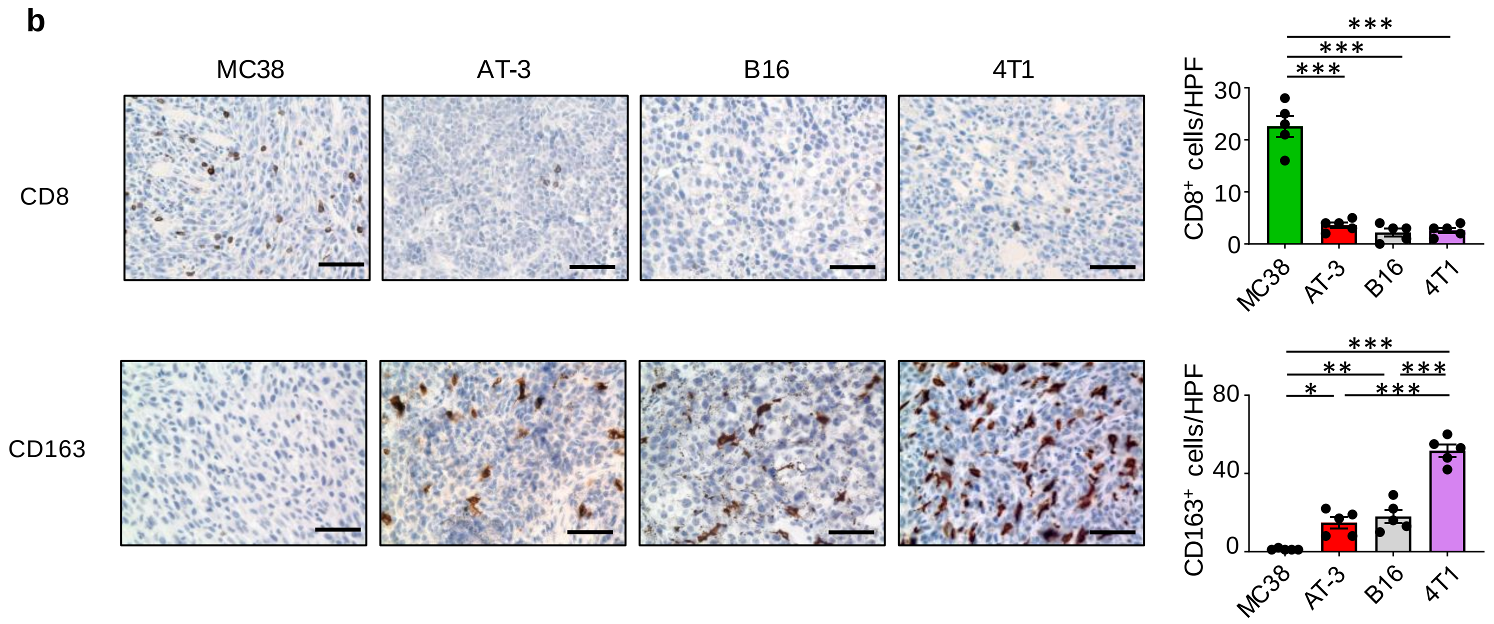

Supplementary Fig. 1 Sensitivity to PD-L1 blockade correlates with density of $CD8^+$ tumor-infiltrating lymphocytes.

a Tumor growth curves (mean) in mice treated with isotype control antibody (Ab) (Isotype Ab) or anti-PD-L1 Ab (αPD-L1). n = 5 mice per group. AT-3 and 4T1 cells were injected into 4th mammary fat pad and MC38 and B16 were subcutaneously. Anti-PD-L1 Ab (10F.9G2) or rat IgG2b (LTF-2) Ab was injected every third day at a dose of 200 μg/mice for four times from the day when tumors reached 50-100 mm^3 in size.

b Representative images of immunohistochemistry for CD8 and CD163 in each tumor harvested 14 days after tumor inoculation. Scale bars,100 μm. Data panels show mean numbers of CD8 or CD163 positive cells per each high-power field (HPF) within 5 different areas for each tumor.

a, b $*p < 0.05$, $**p < 0.01$, $***p < 0.001$ by a two-tailed *t*-test (**a**) or a one-way ANOVA with Tukey's multiple comparisons (**b**). Mean ± SEM. Data shown are representative of two (**b**) or three (**a**) independent experiments. Source data are provided as a Source Data file.

Supplementary Fig. 2

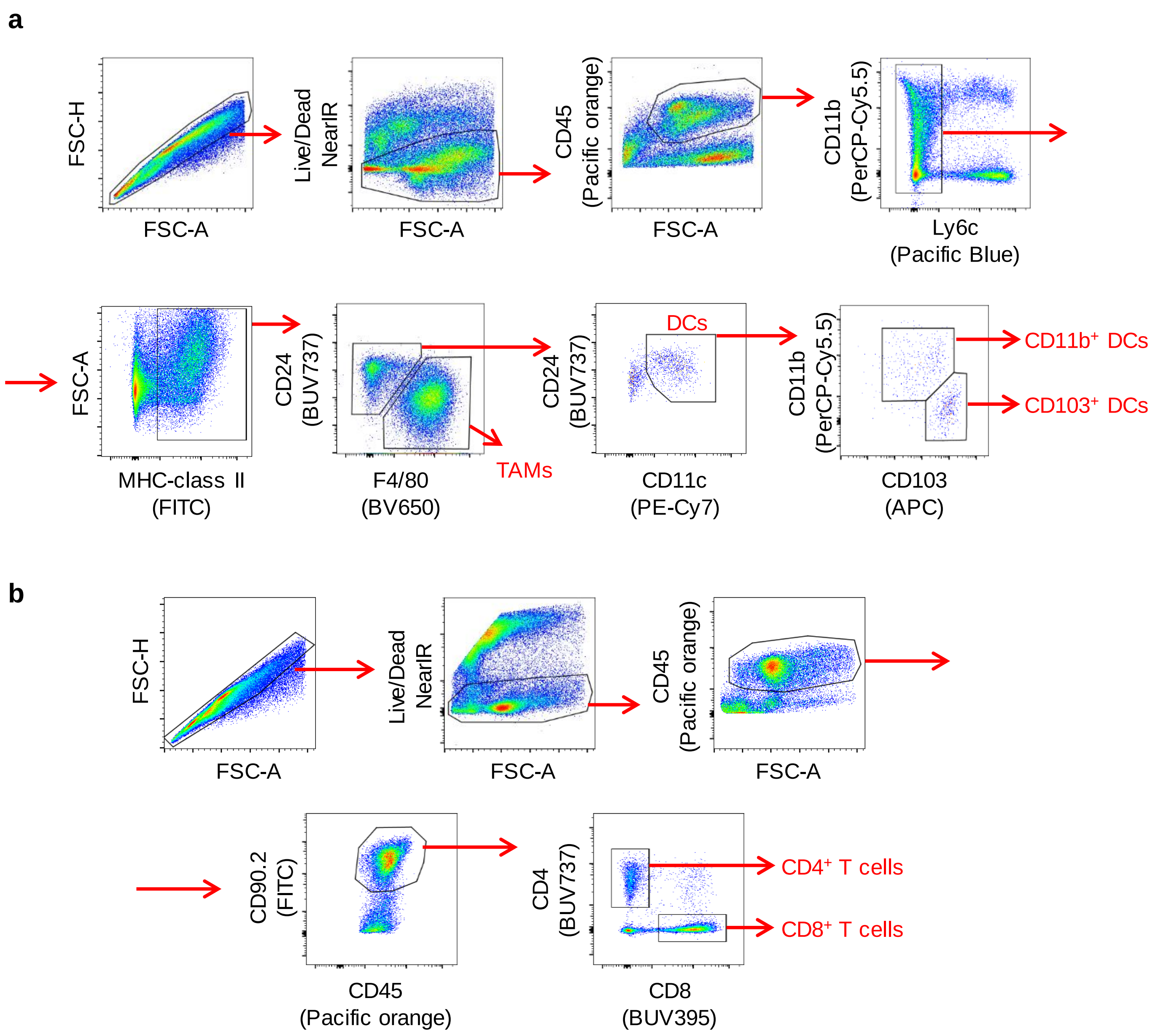

Supplementary Fig. 2 Gating strategy for identifying tumor-residing dendritic cells (DC) and tumor associated macrophages (TAM) (**a**), and T cells (**b**).

a Following gating to include singlets (FSC-H v.s. FSC-A) and live cells by Live/DEAD Fixable NearIR Dead Cell stain, tumor-infiltrating cells were gated for CD45^{+} cells. DCs were identified as Ly6c^{-}/MHC class II^{+}/CD24^{+}/CD11c^{+} cells among CD45^{+} live cells. Based on CD11b and CD103 expression, DCs were divided into two subsets, CD11b^{+} DCs and CD103^{+} DCs. TAMs were identified by expression of CD24^{-} and F4/80^{+} within Ly6c^{-}/MHC class II^{+} cells presented on Fig. 1b, c, Fig. 3b, e, and f, Fig. 4b, d, e, Fig. 5b, Fig. 9 c, d, i, j, Supplementary Fig. 3 a, b and Supplementary Fig. 19 c, d.

b Following gating to include singlets (FSC-H v.s. FSC-A) and live cells by Live/DEAD Fixable NearIR Dead Cell stain, tumor-infiltrating cells were gated for CD45^{+} cells. T cells were identified as CD4^{+} CD90.2^{+} or CD8^{+} CD90.2^{+} cells.

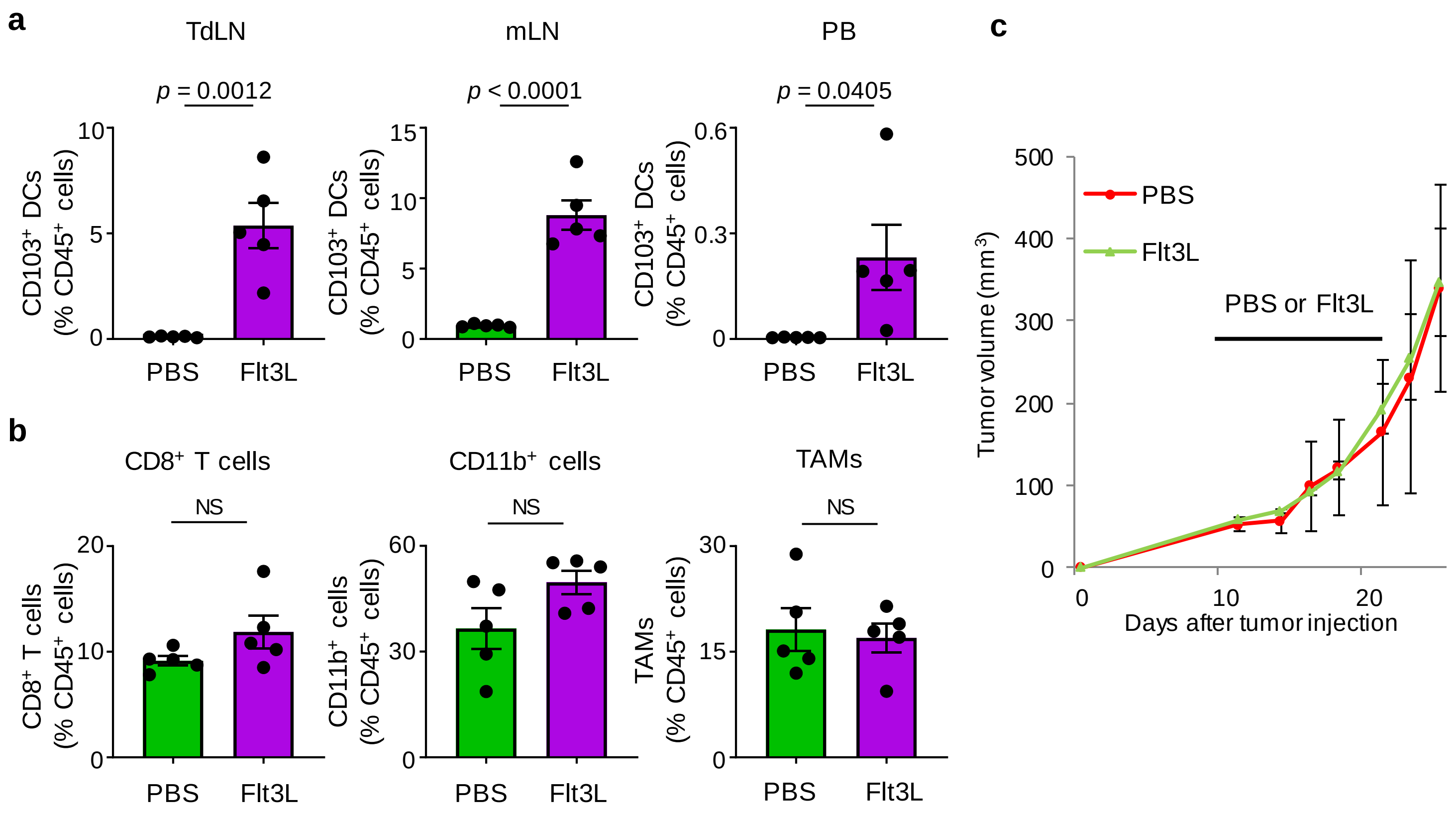

Supplementary Fig. 3 *In situ* administration of Flt3L induces systemic increase of CD103$^+$ DCs. Related to Figure 1b.

a, b Mice bearing AT-3 tumors were treated with intratumoral injection of PBS or Flt3L (30 µg/dose) daily for 9 days. Tumor-draining lymph nodes (TdLN), mesenteric lymph nodes (mLN) peripheral blood (PB) (**a**) and tumors (**b**) were harvested 1 day after completion of PBS or Flt3L injection.

a Frequency of CD103$^+$ DCs (Ly6c$^-$ class II$^+$ CD11c$^+$ CD24$^+$ F4/80$^-$ CD103$^+$) among CD45$^+$ cells in TdLN, mLN and PB of AT-3 tumor bearing mice treated with PBS or Flt3L. n = 5 mice per group.

b Frequency of CD8$^+$ T cells, CD11b$^+$ cells, and Ly6c$^-$ class II$^+$ CD24$^-$ F4/80$^+$ tumor associated macrophages (TAMs) among CD45$^+$ cells in AT-3 tumors. n = 5 mice per group.

c Tumor growth curves (mean) in AT-3 tumor bearing mice treated with PBS and Flt3L. n = 5 mice per group. NS: not significant. Statistical significance was determined by a two-tailed *t*-test (**a, b**). Mean ± SEM. Source data are provided as a Source Data file.

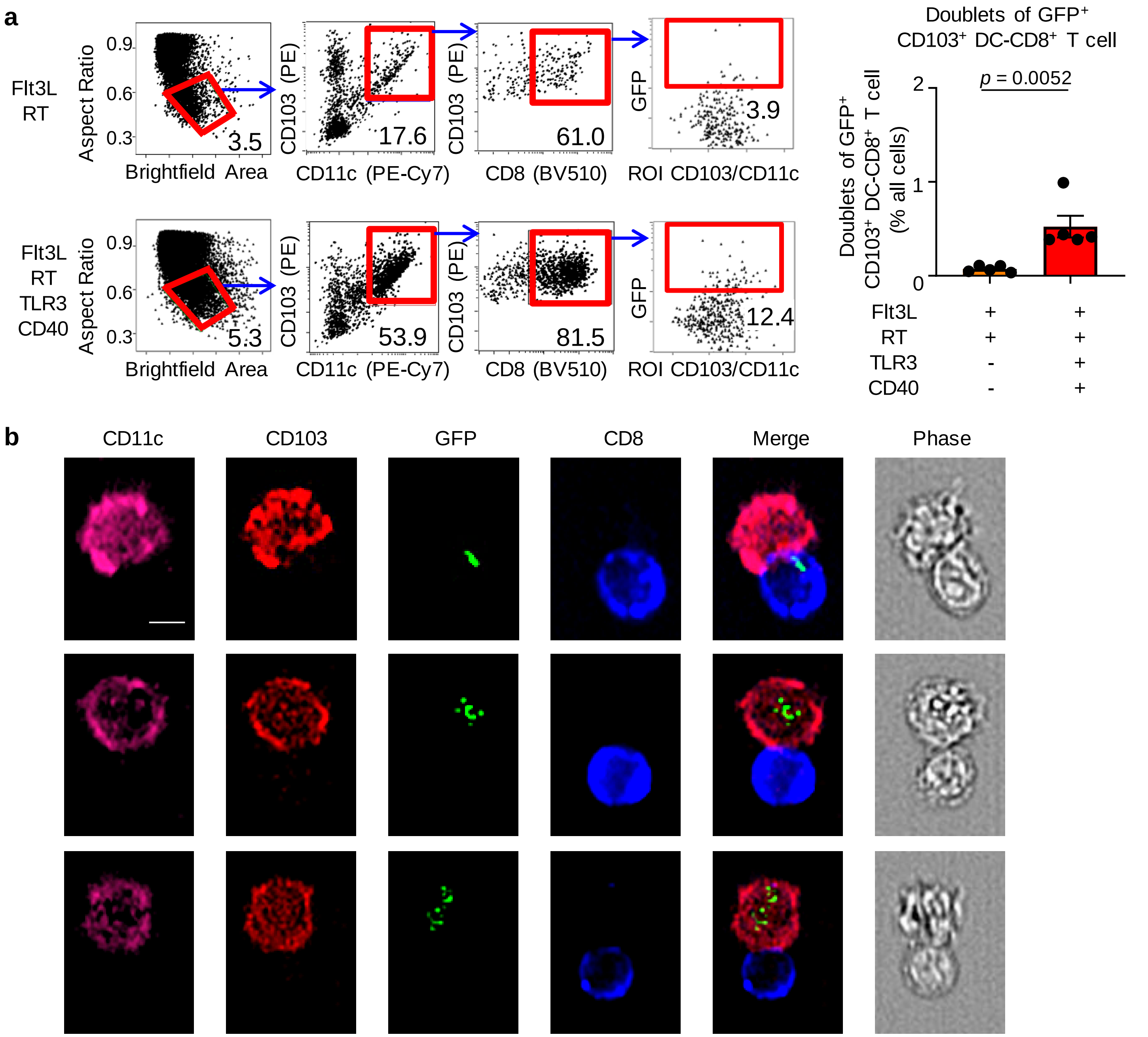

Supplementary Fig. 4 *In situ* TLR3/CD40 stimulation following Flt3L administration and radiotherapy (RT) increases doublets of tumor-associated antigen loaded CD103+ DC and CD8+ T cell in tumor-draining lymph nodes (TdLN). Related to Figure 1e.

a, b Mice bearing AT-3 tumors expressing GFP (AT-3-GFP) were treated with *in situ* injections of TLR3/CD40 agonists or phosphate-buffered saline (PBS) following Flt3L administration and RT, and TdLN were harvested after 24h.

a Representative flow cytometric plots identifying doublets of GFP+ CD103+ DC-CD8+ T cell in TdLN. Numbers denote percent gated cells. Data panel shows frequency of doublets of GFP+ CD103+ DC-CD8+ T cell out of all cells in TdLN. Doublets of CD103+ DC-CD8+ T cell were identified based on the expression of CD103, CD11c and CD8. To identify doublets of GFP+ CD103+ DC-CD8+ T cell, a region of interest (ROI) was created based on the location of the CD103 and CD11c expression, and the GFP fluorescence intensity restricted to that ROI for each doublet was then quantified. n = 5 mice per group. Statistical significance was determined by a two-tailed *t*-test. Mean ± SEM.

b Representative images of doublets of GFP+ CD103+ DC-CD8+ T cell from (**a**). Images shown are from representative of two independent experiments. All panels are the same magnification, scale bar = 10 µm. Source data are provided as a Source Data file.

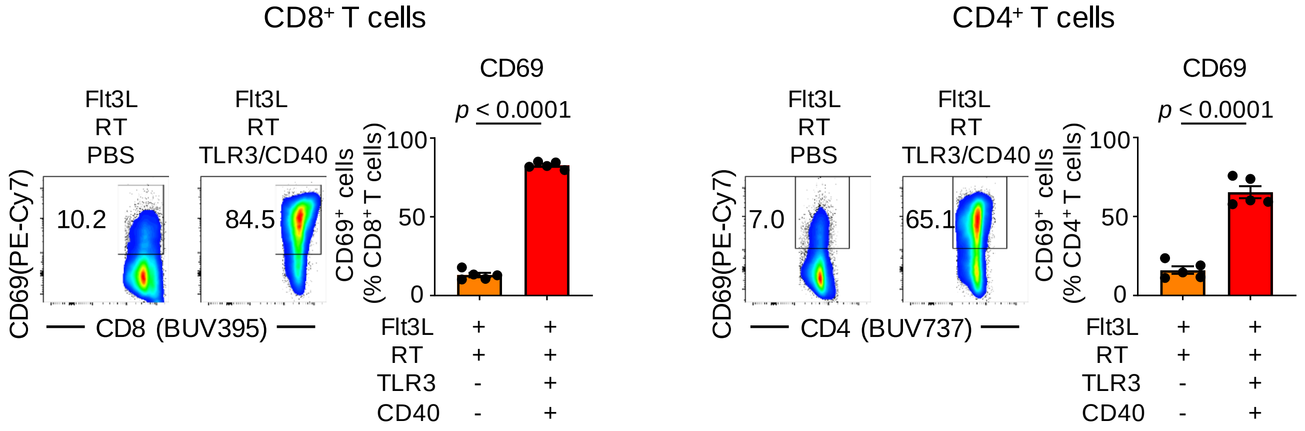

Supplementary Fig. 5 *In situ* TLR3/CD40 stimulation of induced CD103+ DCs triggers activation of T cells in tumor-draining lymph nodes (TdLN). Related to Figure 1e.

Representative flow cytometric plots showing CD69 positive cells in CD8+ T cells (left) and CD4+ T cells (right) in TdLN from AT-3 tumor-bearing C57BL/6 mice treated with Flt3L+RT or Flt3L+RT+TLR3/CD40 stimulation (ISIM: *in situ* immunomodulation). Data panels show percentage of CD69 positive cells in CD8+ T cells and CD4+ T cells in TdLN. n = 5 mice per group. TdLN were harvested 1 day after *in situ* TLR3/CD40 stimulation. Numbers denote percent CD69+ cells. Statistical significance was determined by a two-tailed *t*-test. Mean ± SEM. Source data are provided as a Source Data file.

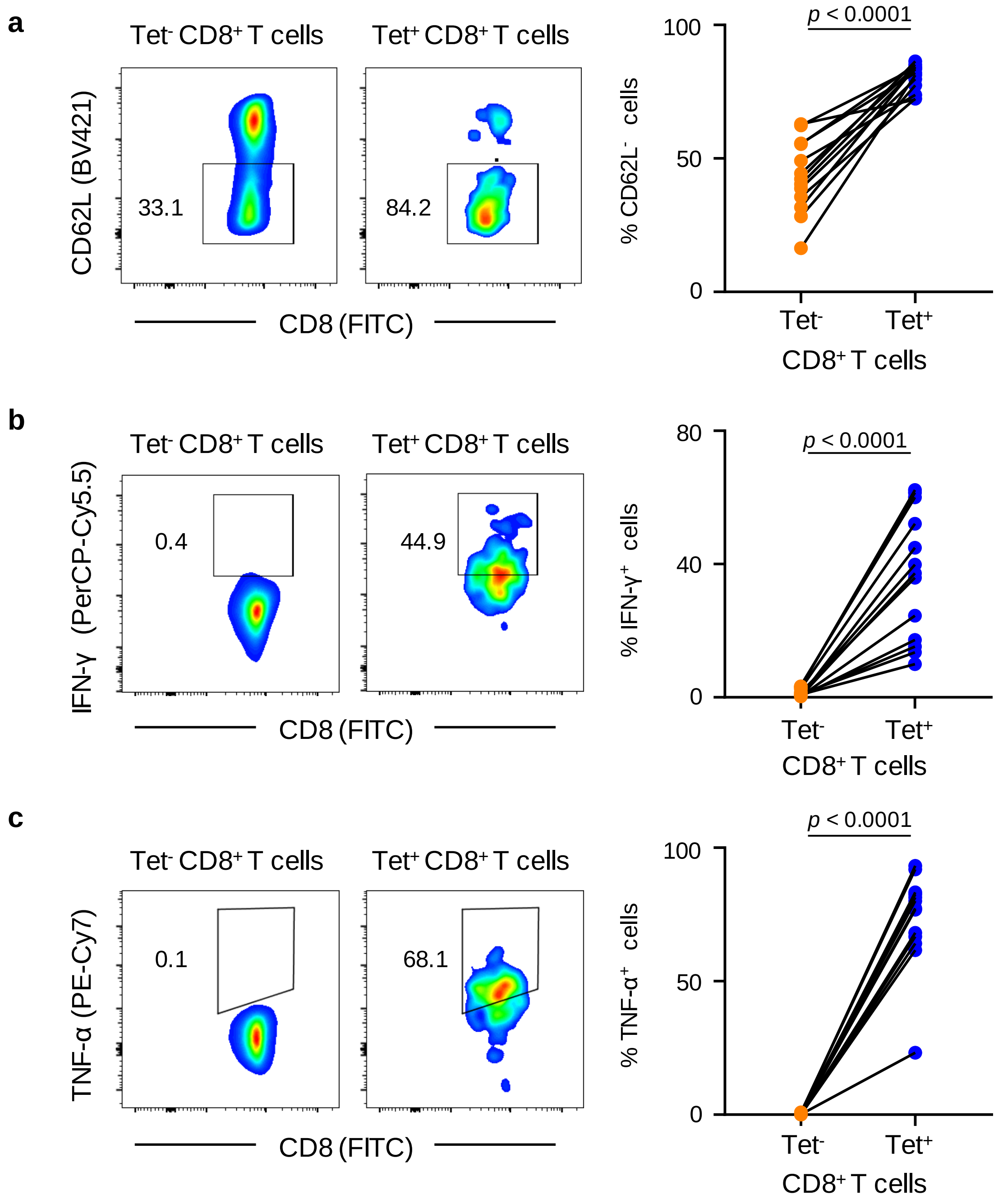

Supplementary Fig. 6 Tumor-specific $CD8^+$ T cells in tumor-draining lymph nodes (TdLN) generated by *in situ* immunomodulation (ISIM) exhibit an effector phenotype and cytokine-producing capacity. Related to Figure 1f.

a-c 4T1 tumor-bearing BALB/c mice treated with ISIM, and TdLN were harvested 1 day after *in situ* TLR3/CD40 stimulation. n = 13 mice per group.

a Representative flow cytometric plots showing CD62L expression in gp70-specific Tet^- (left) or Tet^+ (right) $CD8^+$ T cells in TdLN. Numbers denote percent $CD62L^-$ cells. Data panels show percentage of $CD62L^-$ cells in Tet^- or Tet^+ $CD8^+$ T cells in TdLN.

b, c Representative flow cytometric plots showing IFN-γ (**b**) and TNF-α (**c**) expression in Tet^- (left) or Tet^+ (right) $CD8^+$ T cells in TdLN. Cells harvested from TdLN were co-cultured with AH1 peptide (SPSYVYHQF) in the presence of Brefeldin A for 5hrs before intracellular staining. Numbers denote percent IFN-γ^+ (**b**) and TNF-α^+ (**c**) cells. Data panels show percentage of IFN-γ (**b**) and TNF-α (**c**) positive cells in Tet^- or Tet^+ $CD8^+$ T cells in TdLN. Statistical significance was determined by a paired two-tailed *t*-test (**a-c**). Source data are provided as a Source Data file.

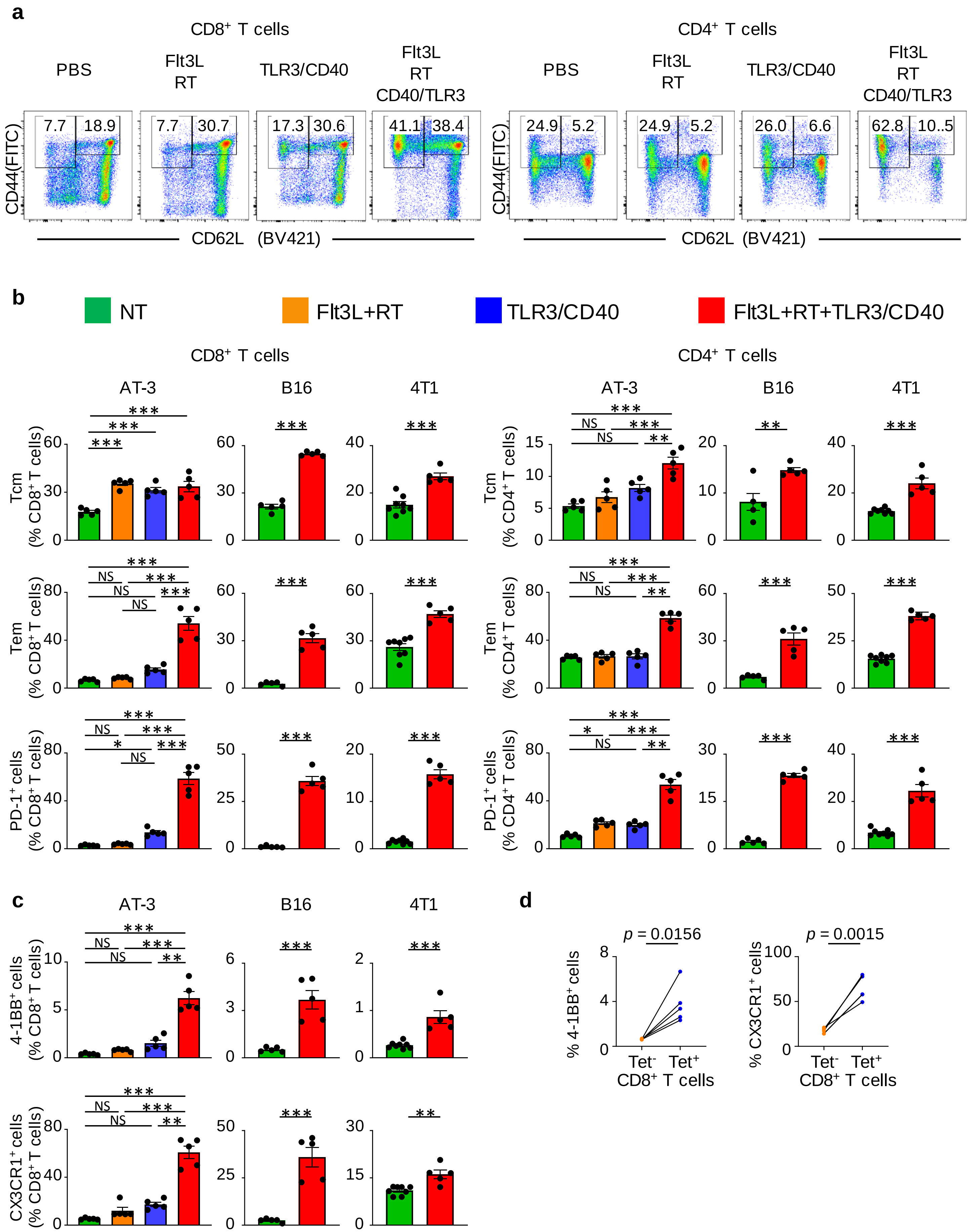
a
CD8+ T cells
CD4+ T cells
PBS
Flt3L RT
TLR3/CD40
Flt3L RT CD40/TLR3
7.7 18.9
7.7 30.7
17.3 30.6
41.1 38.4
24.9 5.2
24.9 5.2
26.0 6.6
62.8 10..5
CD44(FITC)
CD62L (BV421)
b
NT
Flt3L+RT
TLR3/CD40
Flt3L+RT+TLR3/CD40
AT-3
B16
4T1
Tcm (% CD8+ T cells)
Tem (% CD8+ T cells)
PD-1+ cells (% CD8+ T cells)
Tcm (% CD4+ T cells)
Tem (% CD4+ T cells)
PD-1+ cells (% CD4+ T cells)
c
4-1BB+ cells (% CD8+ T cells)
CX3CR1+ cells (% CD8+ T cells)
d
p = 0.0156
p = 0.0015
% 4-1BB+ cells
% CX3CR1+ cells
Tet- Tet+
CD8+ T cells

Supplementary Fig. 7 *In situ* immunomodulation (ISIM) significantly increases circulating effector memory T cells.

a Representative flow cytometric plots showing CD44 and CD62L expression in $CD8^+$ T cells (left) and $CD4^+$ T cells (right) in peripheral blood (PB) of AT-3 tumor-bearing mice in different treatment groups as indicated. RT: radiotherapy. Numbers denote percent effector memory T cells (Tem: $CD44^+$ $CD62L^-$) (left), and central memory T cells (Tcm: $CD44^+$ $CD62^+$) (right).

b Frequency of Tcm, Tem and $PD\text{-}1^+$ subsets among $CD8^+$ T cells (left) and $CD4^+$ T cells (right) in PB of AT-3, B16 or 4T1 tumor-bearing mice in different treatment groups as indicated. n = 8 mice (NT of 4T1) and 5 mice (any other groups).

c Frequency of $4\text{-}1BB^+$ and $CX3CR1^+$ subsets among $CD8^+$ T cells in PB of AT-3, B16 or 4T1 bearing mice in different treatment groups as indicated. n = 8 mice (NT of 4T1) and 5 mice (any other groups).

a-c NS: not significant, * $p < 0.05$, ** $p < 0.01$, ***$p < 0.001$ by a two-tailed *t*-test for comparisons between two groups or a one-way ANOVA with Tukey's multiple comparisons for comparisons of more than two groups.

d Percentage of $4\text{-}1BB^+$ (left) and $CX3CR1^+$ (right) cells in PB gp70-specific Tet^- or Tet^+ $CD8^+$ T cells from ISIM-treated 4T1 tumor-bearing mice. n = 5 mice per group. Statistical significance was determined by a paired two-tailed *t*-test.

a-d PB was harvested 5-7 days after *in situ* TLR3/CD40 stimulation. Mean ± SEM. Data shown are representative of two (**d**) or three (**a-c**) independent experiments. Source data are provided as a Source Data file.

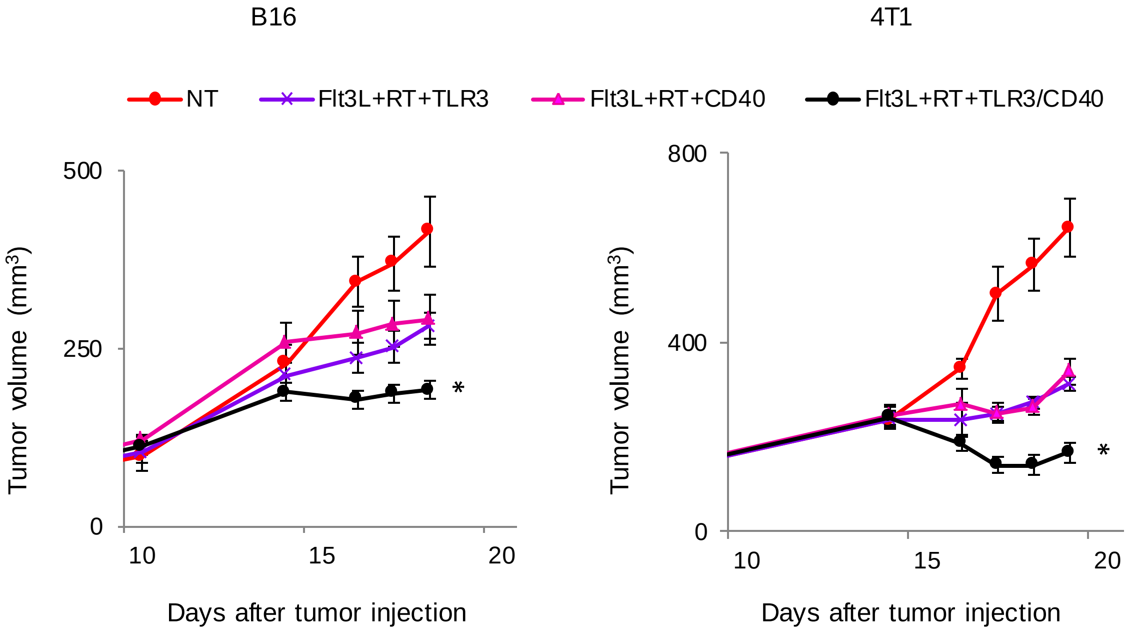

Supplementary Fig. 8 Dual TLR3/CD40 stimulation synergistically enhances antitumor efficacy of *in situ* immunomodulation (ISIM). Related to Figure 4.

Tumor volume curves (mean) in B16 (left) and 4T1 (right) tumor-bearing mice in different treatment as indicated. RT: radiotherapy. B16; n = 6 mice (NT), 7 mice (Flt3L+RT+TLR3), and 8 mice (Flt3L+RT+CD40, Flt3L+RT+TLR3/CD40). 4T1; n = 6 mice in all groups. Mice were treated with PBS or Flt3L daily for 9 consecutive days at day 4-12. RT and TLR3/CD40 agonists were administered at day 13 and day 14, respectively. *p < 0.01 versus any other groups by a two-tailed t-test. Mean ± SEM. Source data are provided as a Source Data file.

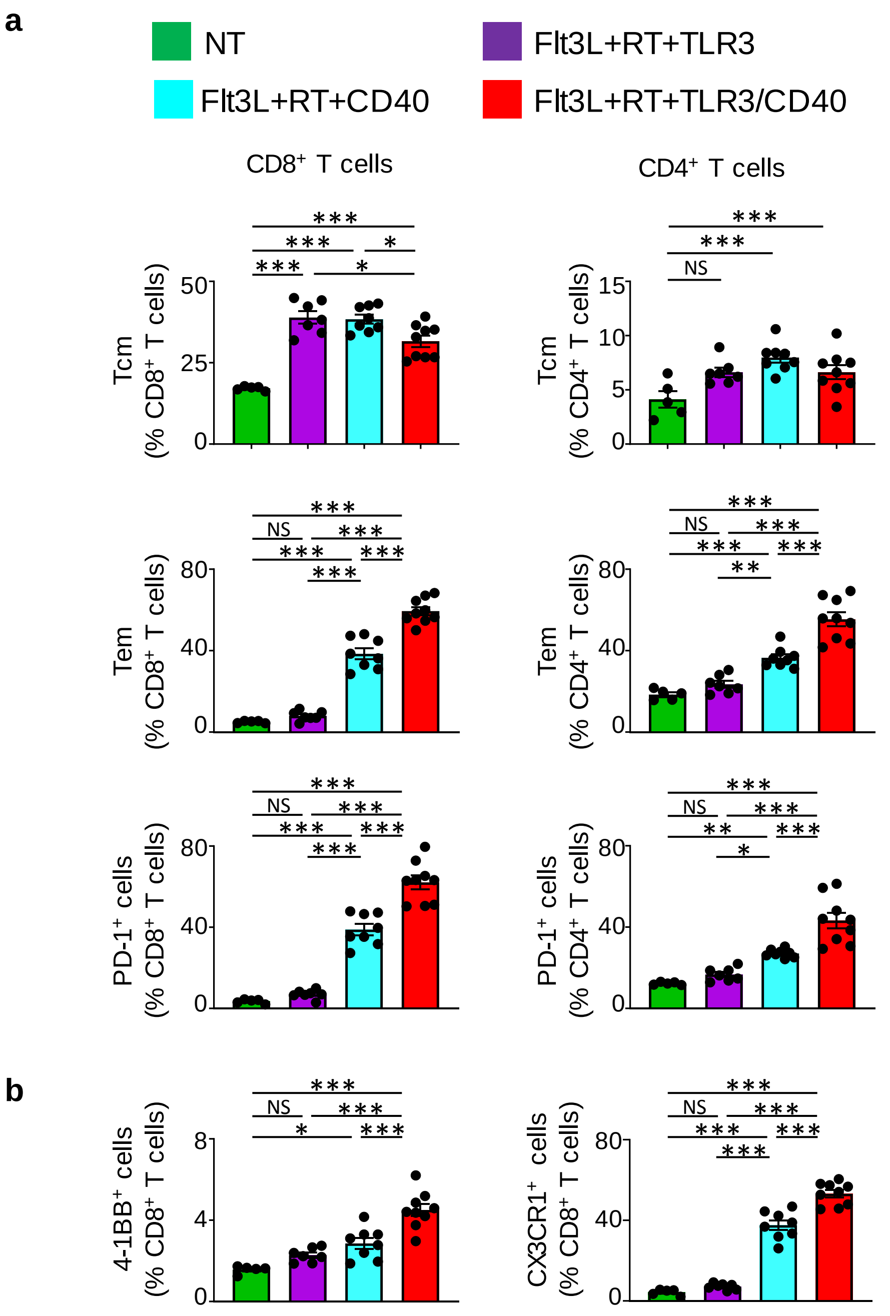

Supplementary Fig. 9 CD40 stimulation of Flt3L-induced dendritic cells is required for the generation of effector memory CD4+ and CD8+ T cells and their upregulation of PD-1, 4-1BB and CX3CR1. Related to Figure 4.

a Frequency of effector memory T cells (Tem: CD44+ CD62L-), central memory T cells (Tcm: CD44+ CD62+), and PD-1 positive cells among CD8+ T cells (left) and CD4+ T cells (right) in peripheral blood (PB) of AT-3 tumor-bearing mice in different treatment groups as indicated. n = 5 mice (NT), 7 mice (Flt3L+RT+TLR3), 8 mice (Flt3L+RT+CD40), and 9 mice (Flt3L+RT+TLR3/CD40).

b Frequency of 4-1BB+ and CX3CR1+ subsets among CD8+ T cells in PB of AT-3 tumor-bearing mice in different treatment groups as indicated. n = 5 mice (NT), 7 mice (Flt3L+RT+TLR3), 8 mice (Flt3L+RT+CD40), and 9 mice (Flt3L+RT+TLR3/CD40).

a, b PB was harvested 7 days after *in situ* TLR3/CD40 stimulation. NS: not significant, $*p < 0.05$, $**p < 0.01$, $***p < 0.001$ by a one-way ANOVA with Tukey's multiple comparisons. Mean ± SEM. Data shown are representative of three independent experiments. Source data are provided as a Source Data file.

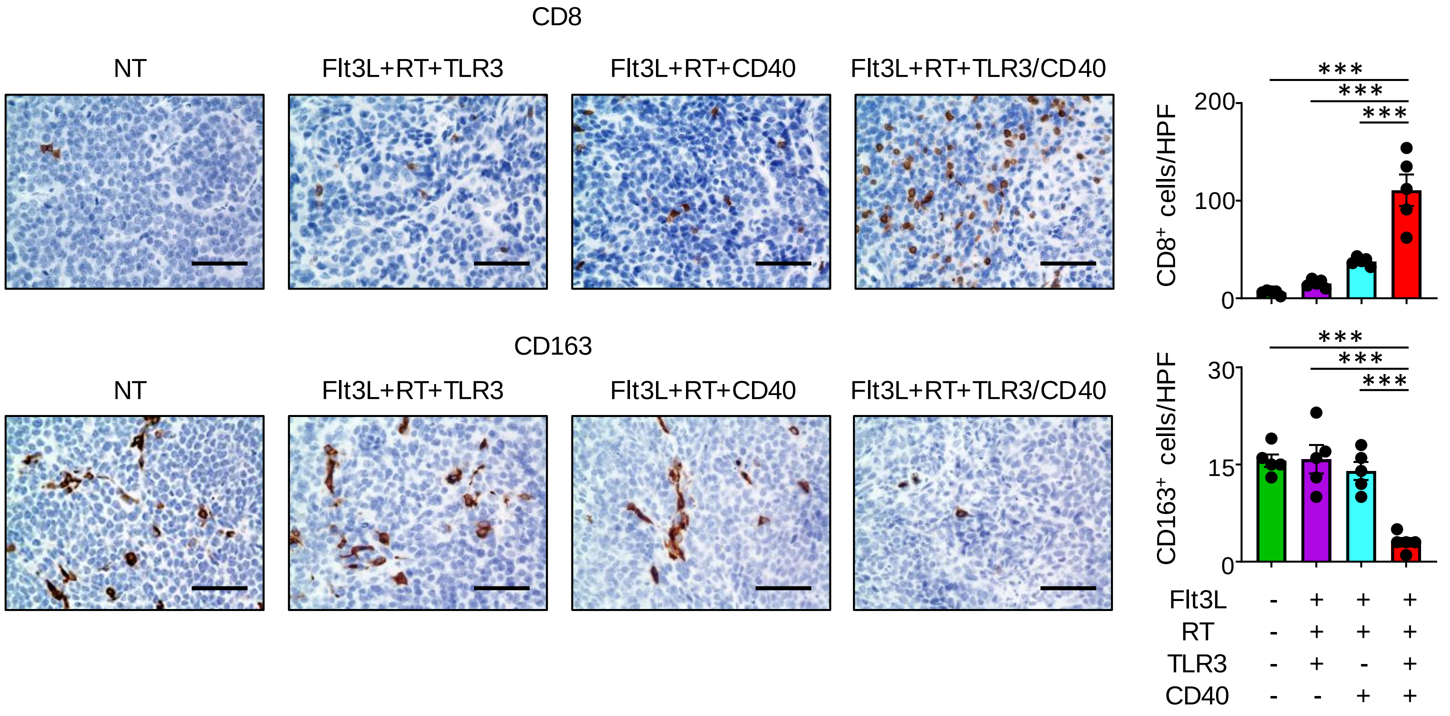

Supplementary Fig. 10 *In situ* dual TLR3/CD40 stimulation of Flt3L-induced DCs synergistically remodels poorly T-cell infiltrated tumors. Related to Figure. 4.

Representative images of immunohistochemistry for CD8 and CD163 in AT-3 tumors from mice in different treatment groups as indicated. Scale bars,100 µm. Data panels show mean numbers of CD8 and CD163 positive cells per each high-power field (HPF) within 5 different areas for each treatment. *** $p < 0.001$ by a one-way ANOVA with Tukey's multiple comparisons. Mean ± SEM. Source data are provided as a Source Data file.

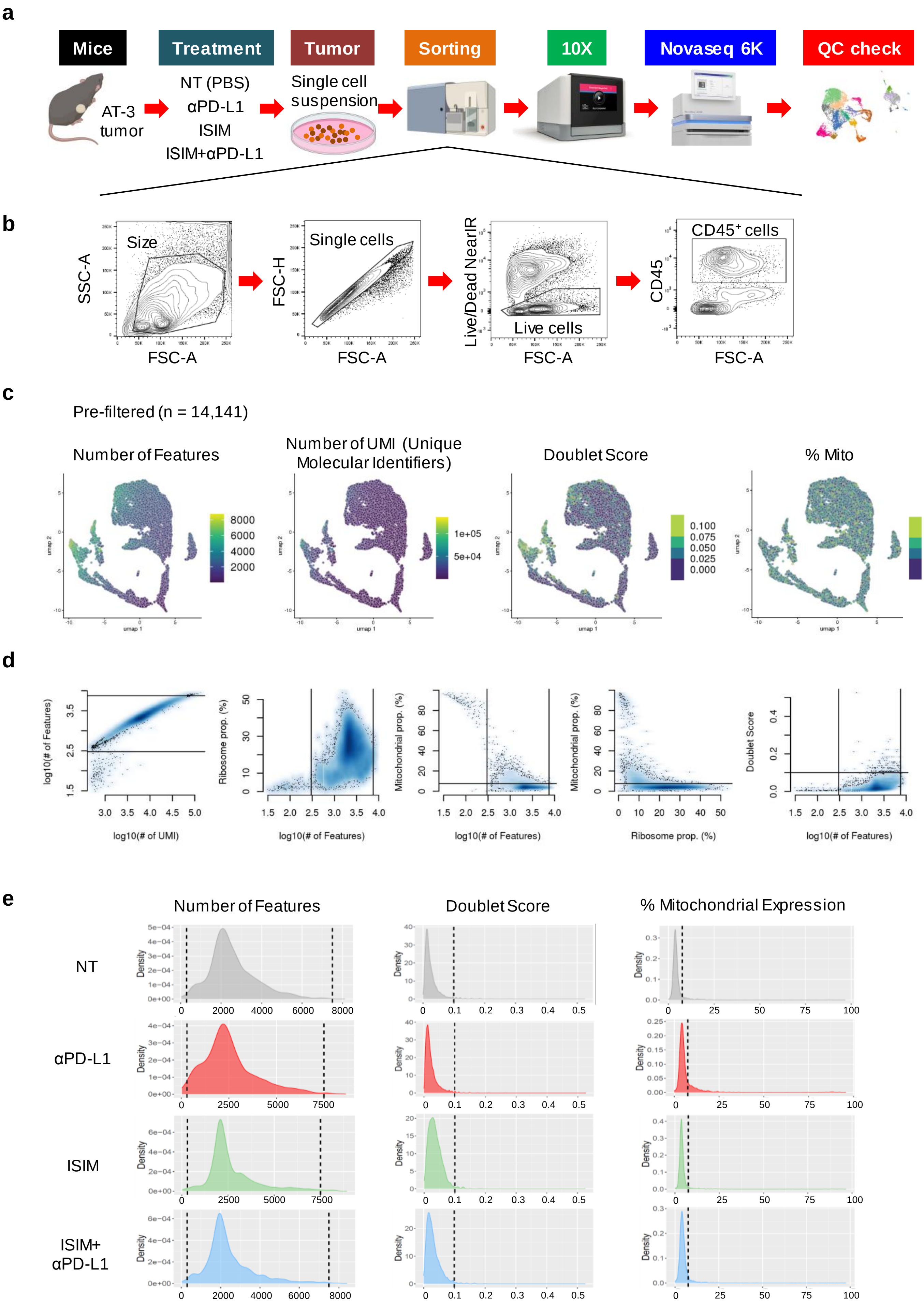

a
Mice
Treatment
Tumor
Sorting
10X
Novaseq 6K
QC check
AT-3 tumor
NT (PBS)
αPD-L1
ISIM
ISIM+αPD-L1
Single cell suspension
b
Size
SSC-A
FSC-A
Single cells
FSC-H
Live/Dead NearIR
Live cells
CD45
CD45⁺ cells
c
Pre-filtered (n = 14,141)
Number of Features
Number of UMI (Unique Molecular Identifiers)
Doublet Score
% Mito
umap 1
umap 2
d
log10(# of Features)
log10(# of UMI)
Ribosome prop. (%)
Mitochondrial prop. (%)
Doublet Score
e
Number of Features
Doublet Score
% Mitochondrial Expression
NT
αPD-L1
ISIM
ISIM+ αPD-L1
Density

Post-filtered (n = 12,228)

f

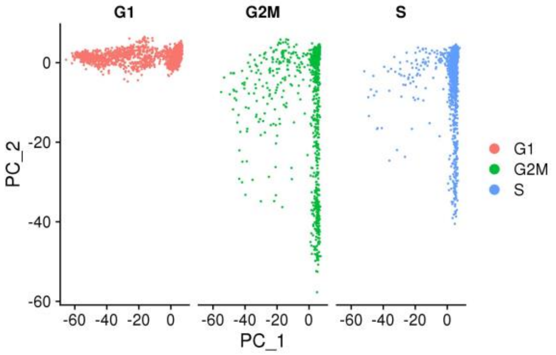

g

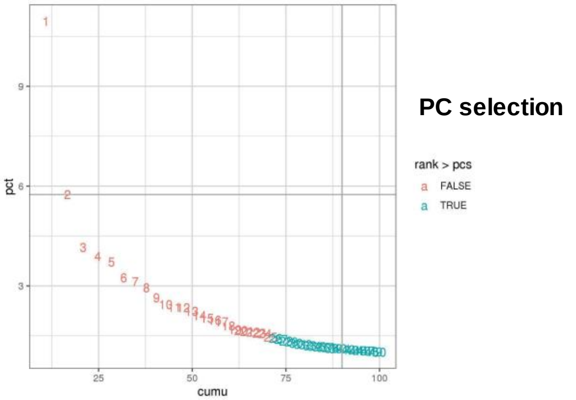

h

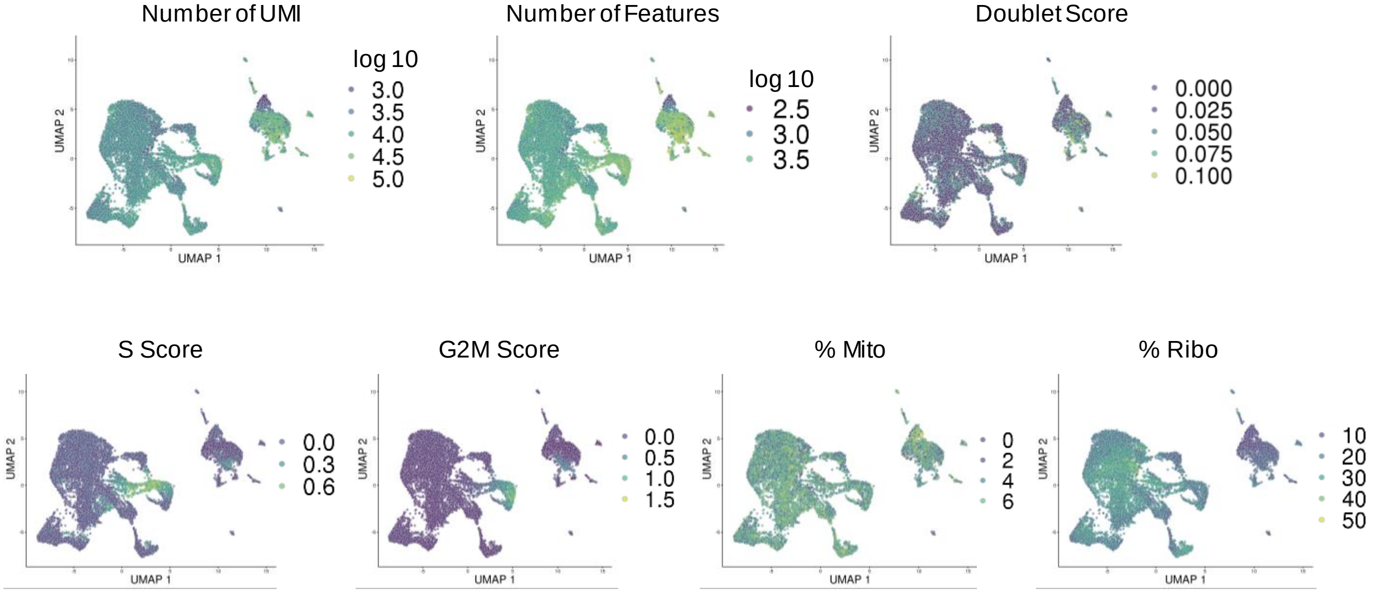

Supplementary Fig. 11 scRNAseq quality assessment. Related to Figure 6-8.
a Overview of the experimental workflow. ISIM: *in situ* immunomodulation. **b** Flow cytometry and sorting of live $CD45^{+}$ tumor-infiltrating single cells in AT-3 tumor-bearing mice. **c** Doublet assessment was performed using a combination of outlier feature expression and quantitative scoring performed via scrublet[69]. For visualization purposes, unfiltered expression counts were log normalized and PCA applied to the top 100 most variable genes prior to non-linear reduction (UMAP) on the top 10 PCs. UMAP plots showing total expression features, unique counts, mitochondrial expression and doublet scores across cells. **d** Scatterplots depicting total features, counts, doublet scores, mitochondrial content and ribosomal content across all cells prior to filtering. Filtering thresholds applied are shown. **e** G2M/S scoring was applied to post-filtered, normalized expression and PCA performed. PCA plot showing cells annotated by cell cycle phase. **f** Distributions of features (left), doublet scores (middle) and mitochondrial content for each sample. Filtering thresholds utilized to remove cells with outlier features (< 300, > 7500), doublet detection (> 0.1) and mitochondrial content (> 7.5%) are shown. **g** Elbow plot of PCA (top 50 components). PC selection for downstream analysis was limited to those components accounting for at least 0.1% of total variation (n = 25). **h** UMAP representations of all post-filtered, and post-normalized cells showing total unique counts, features, doublet scores, G2M/S phase scores, mitochondrial and ribosomal scores.

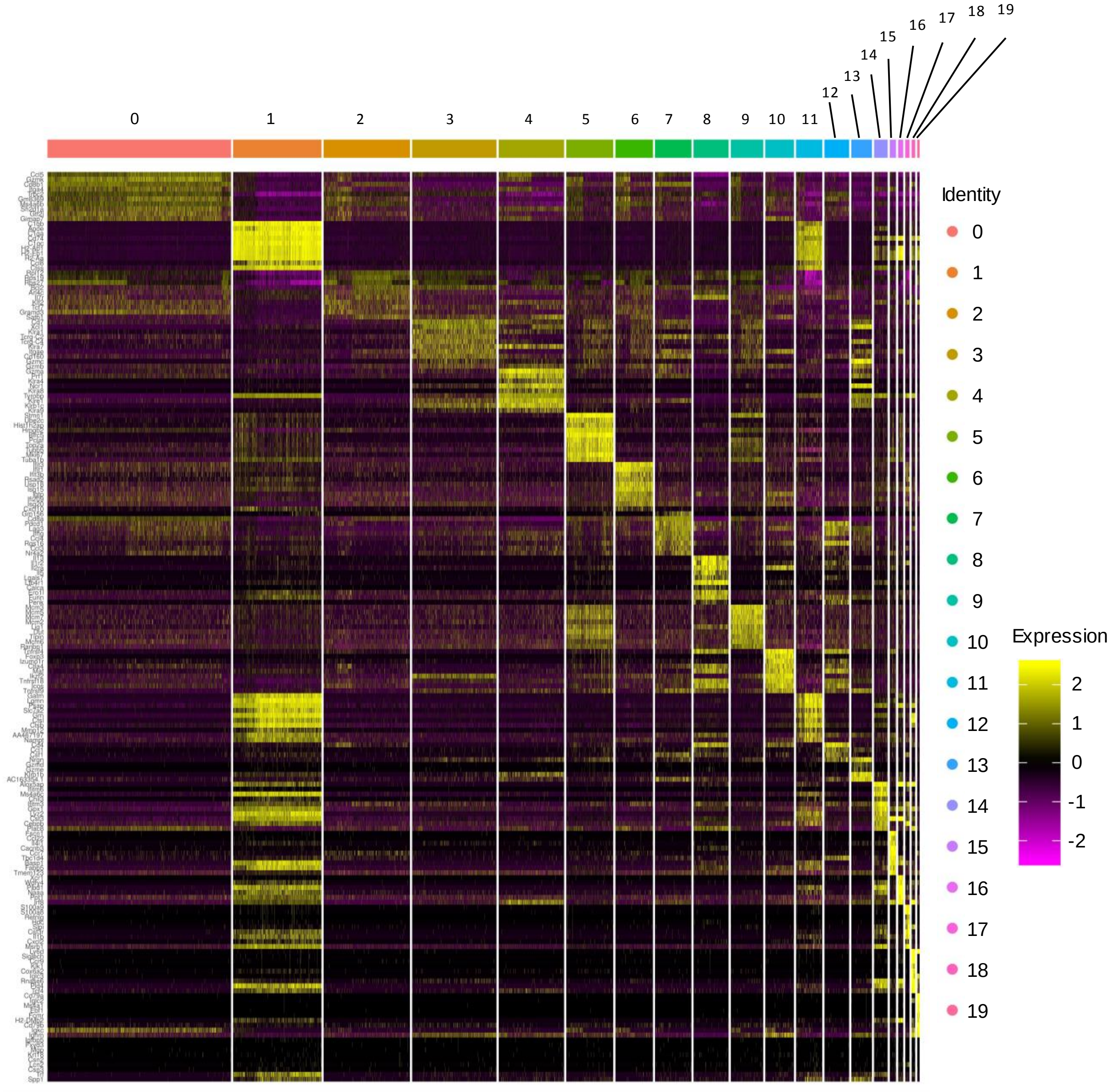

Supplementary Fig. 12. Analysis of intratumoral immune cells by scRNAseq. Related to Figure 6.

Heatmap of all 12,228 cells showing the expression levels of the 10 most discriminative genes per cell type (in rows) across all the identified cell populations (in columns). Gene expression in each clusters are also listed in Supplementary Data 1. Color-code layout: scale of purple to yellow; from lowest expression to highest expression.

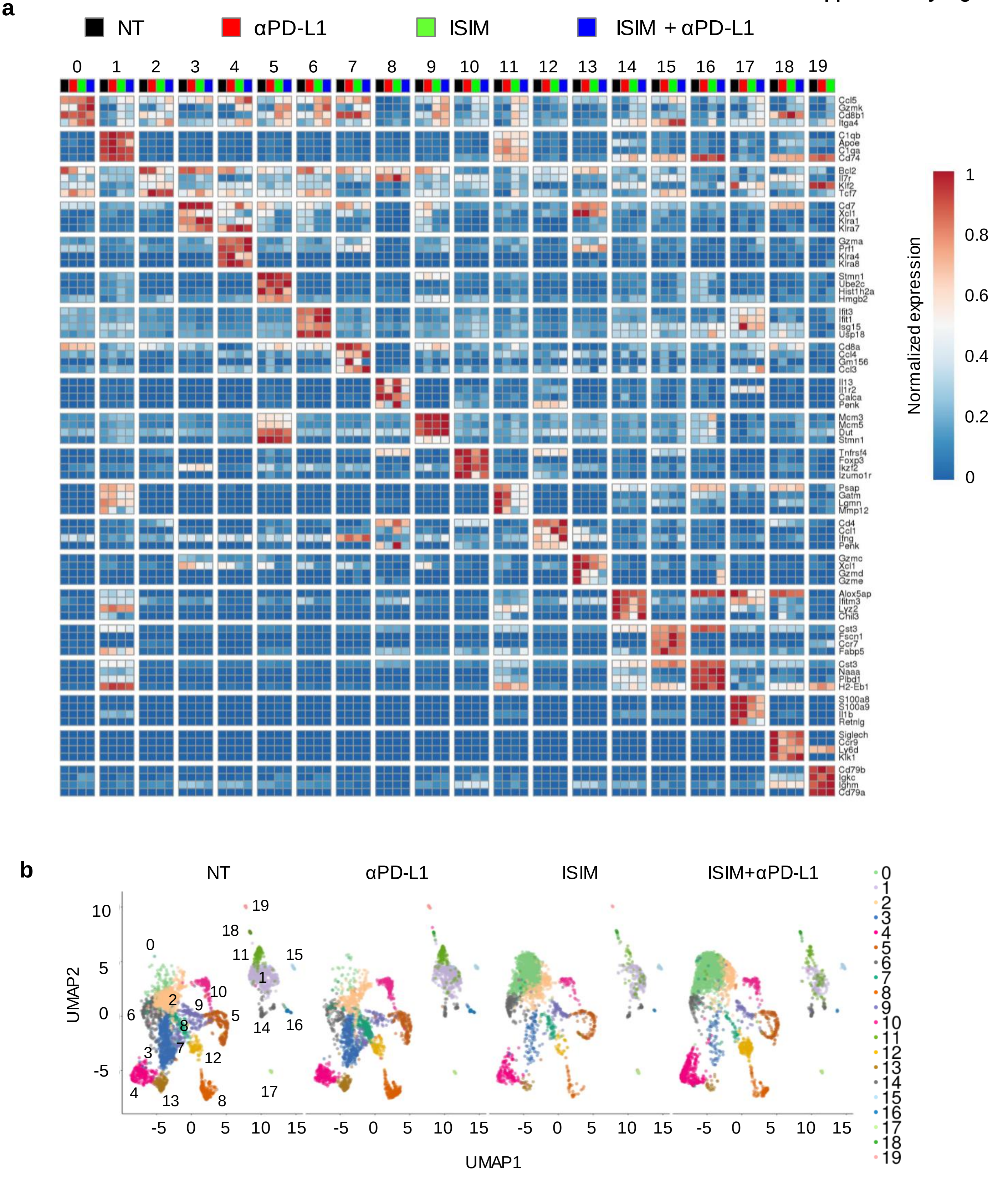

Supplementary Fig. 13 Identification of intratumoral immune cell populations by scRNAseq. Related to Figure 6.

a Heatmap displaying normalized expression of top four genes in each cluster of AT-3 tumor-infiltrating live CD45^{+} cells from mice treated with PBS+isotype Ab (NT), PBS+anti-PD-L1 Ab (αPD-L1), *in situ* immunomodulation (ISIM)+isotype Ab (ISIM), or ISIM+anti-PD-L1 Ab (ISIM+αPD-L1).

b UMAP plots of annotated clusters in different treatments as indicated.

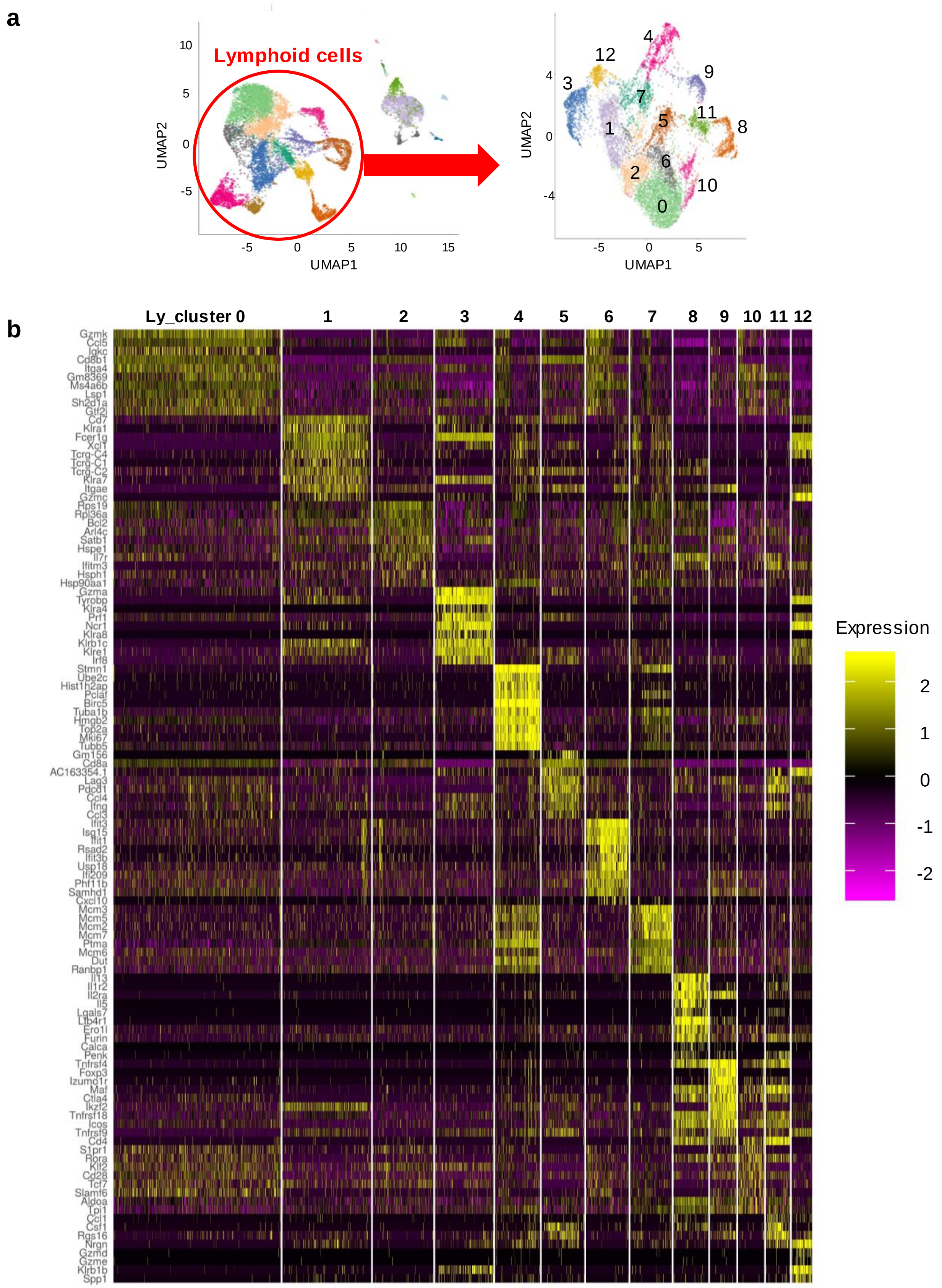

Supplementary Fig. 14 Analysis of tumor-infiltrating lymphoid cells by scRNAseq. Related to Figure 7, 8.
a UMAP plots from merged treatment data of exclusively intratumoral lymphoid cells.
b Heat map of top10 differentially expressed genes in each lymphoid cluster. The columns correspond to the cells; the rows correspond to the genes. Color-code layout: scale of purple to yellow; from lowest expression to highest expression. Gene expression in each clusters are also listed in Supplementary Data 3.

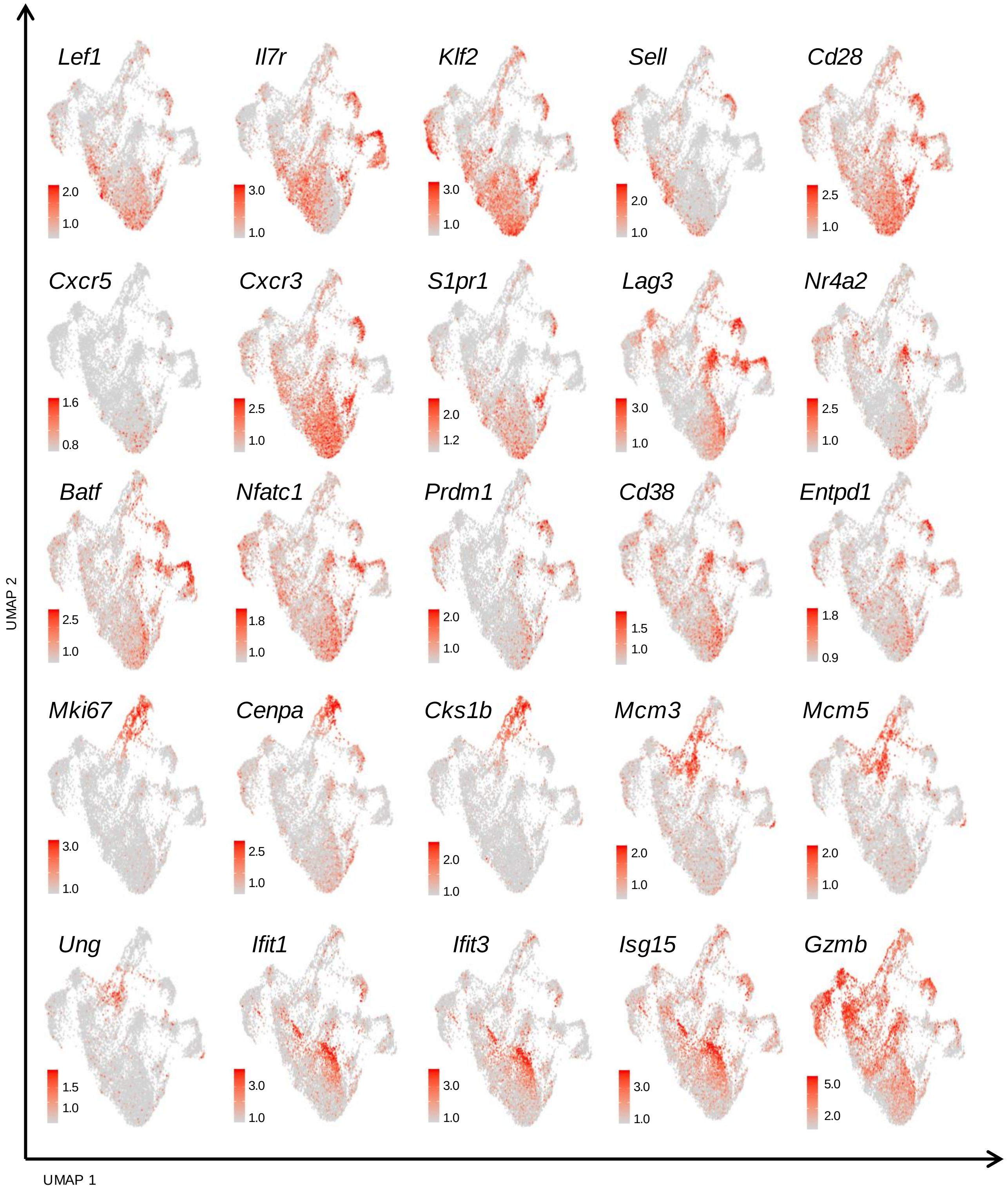

Supplementary Fig. 15 Expression plots of indicated genes in lymphoid cell clusters. Related to Figure 7, 8.

Expression of indicated genes in lymphoid clusters illustrated in UMAP plots. Expression levels are color-coded: gray, not expressed; orange, expressed.

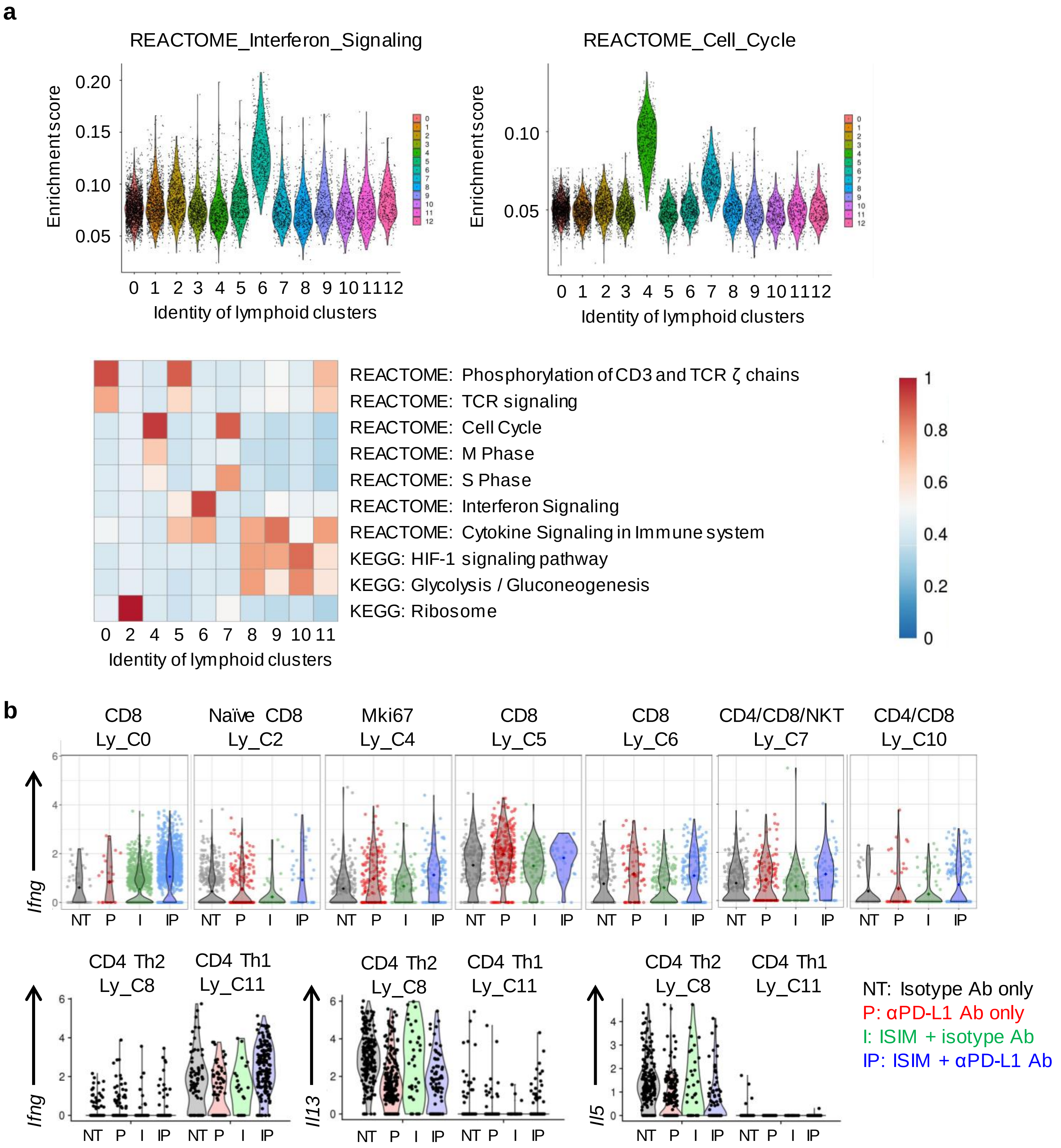

Supplementary Fig. 16 Gene expression and pathway enrichment of tumor-infiltrating lymphoid cell populations by scRNAseq. Related to Figure 7, 8.
a Violin plots of gene set enrichment analysis (GSEA) in each lymphoid cluster. REACTOME : Interferon Signaling and Cell Cycle are shown (upper). Heatmap of GSEA (REACTOME : Phosphorylation of CD3 and TCR ζ chains, TCR signaling, Cell_Cycle, M Phase, S Phase, Interferon Signaling, Cytokine Signaling in Immune system, KEGG : HIF-1 signaling pathway, Glycolysis / Gluconeogenesis, and Ribosome) in lymphoid cluster (Ly_C) 0, 2 and 4-11 (lower).
b Violin plots of *ifng*, *Il13* and *Il15* in each lymphoid cluster in AT-3 tumors treated with PBS+isotype Ab (NT), PBS+anti-PD-L1 Ab (P), *in situ* immunomodulation (ISIM)+isotype Ab (I), or ISIM+anti-PD-L1 Ab (IP).

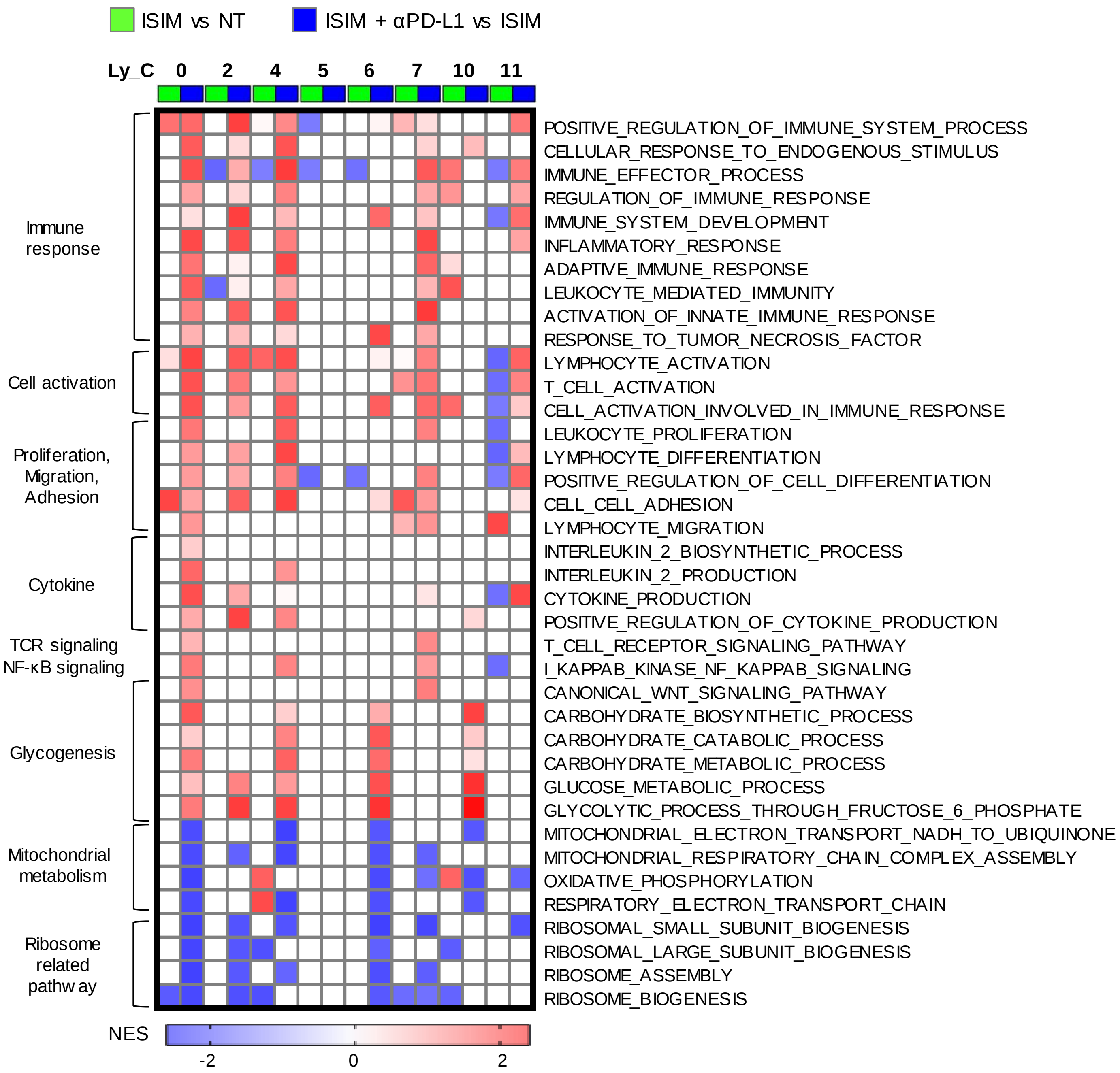

Supplementary Fig. 17 Gene Ontology (GO) enrichment analysis across lymphoid clusters. Related to Figure 7, 8.

Heat map of gene set enrichment analysis (GSEA) of *in situ* immunomodulation (ISIM) versus NT and ISIM+αPD-L1 versus ISIM in Ly_C 0, 2, 4, 5, 6, 7, 10 and 11 showing normalized enrichment score (NES). Gene sets of Gene Ontology (GO) are examined. Only gene sets with Benjamini–Hochberg-adjusted $P < 0.05$ and FDR-q < 0.25 were considered as significantly enriched.

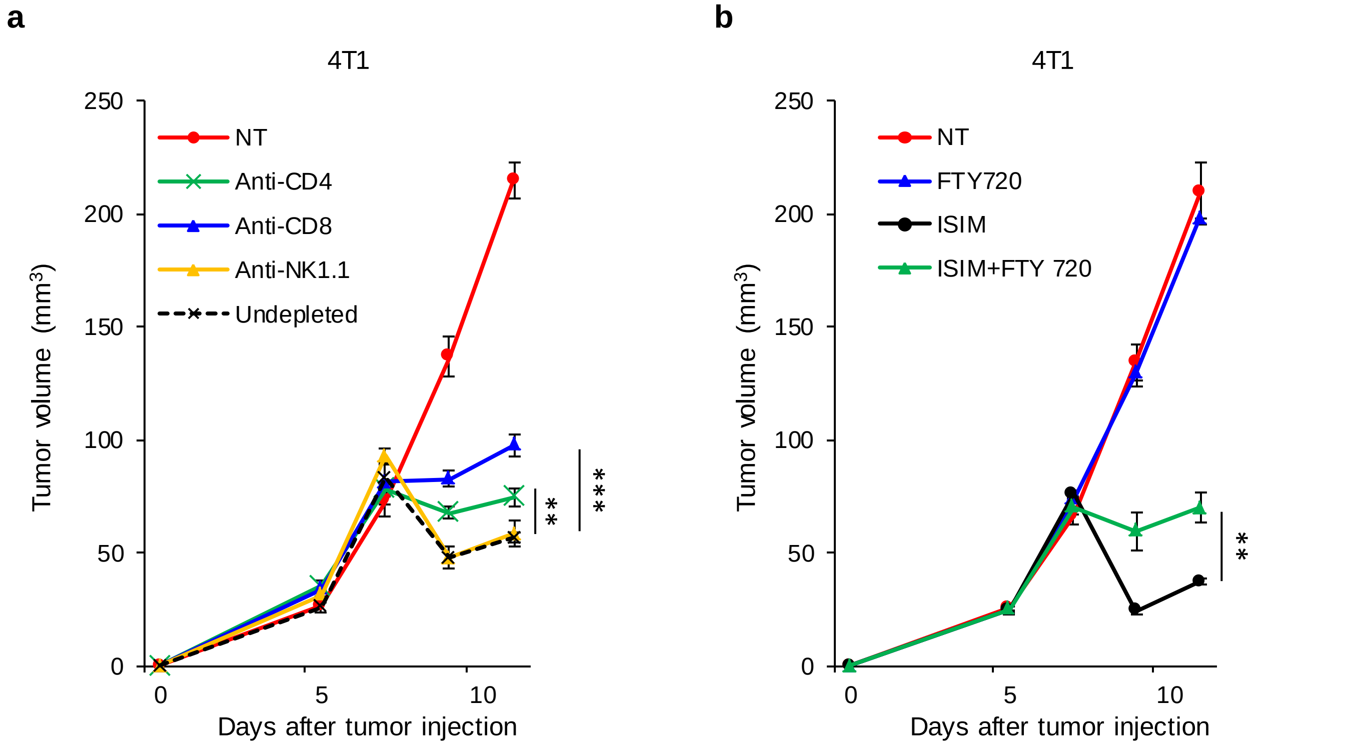

Supplementary Fig. 18 Antitumor efficacy of *in situ* immunomodulation (ISIM) is associated with *de novo* adaptive T-cell immunity. Related to Figure 9b, e.

a Tumor volume curves (mean) in 4T1 tumor-bearing BALB/c mice in different treatment as indicated. n = 6 mice (NT) and 7 mice (Anti-CD4, Anti-CD8, Anti-NK1.1, and Undepleted). For *in vivo* depletion of lymphocytes, 200 μg of anti-CD4, anti-CD8β, anti-NK1.1 Ab, or rat IgG2b were injected intraperitonially every third day for three times from the day when RT was given.

b Tumor volume curves (mean) in 4T1 tumor-bearing BALB/c mice in different treatment as indicated. n = 10 mice in all groups. FTY 720 treatment (20 μg) was initiated one day before RT and was given daily. Mice were treated with PBS or Flt3L daily for 5 consecutive days at day 2 - 6. RT and TLR3/CD40 agonists were administered at day 7 and day 8, respectively.

a, b Data shown are representative of two independent experiments. NS: not significant, $^{**}p < 0.01$, $^{***}p < 0.001$ by a two-tailed *t*-test. Mean ± SEM. Source data are provided as a Source Data file.

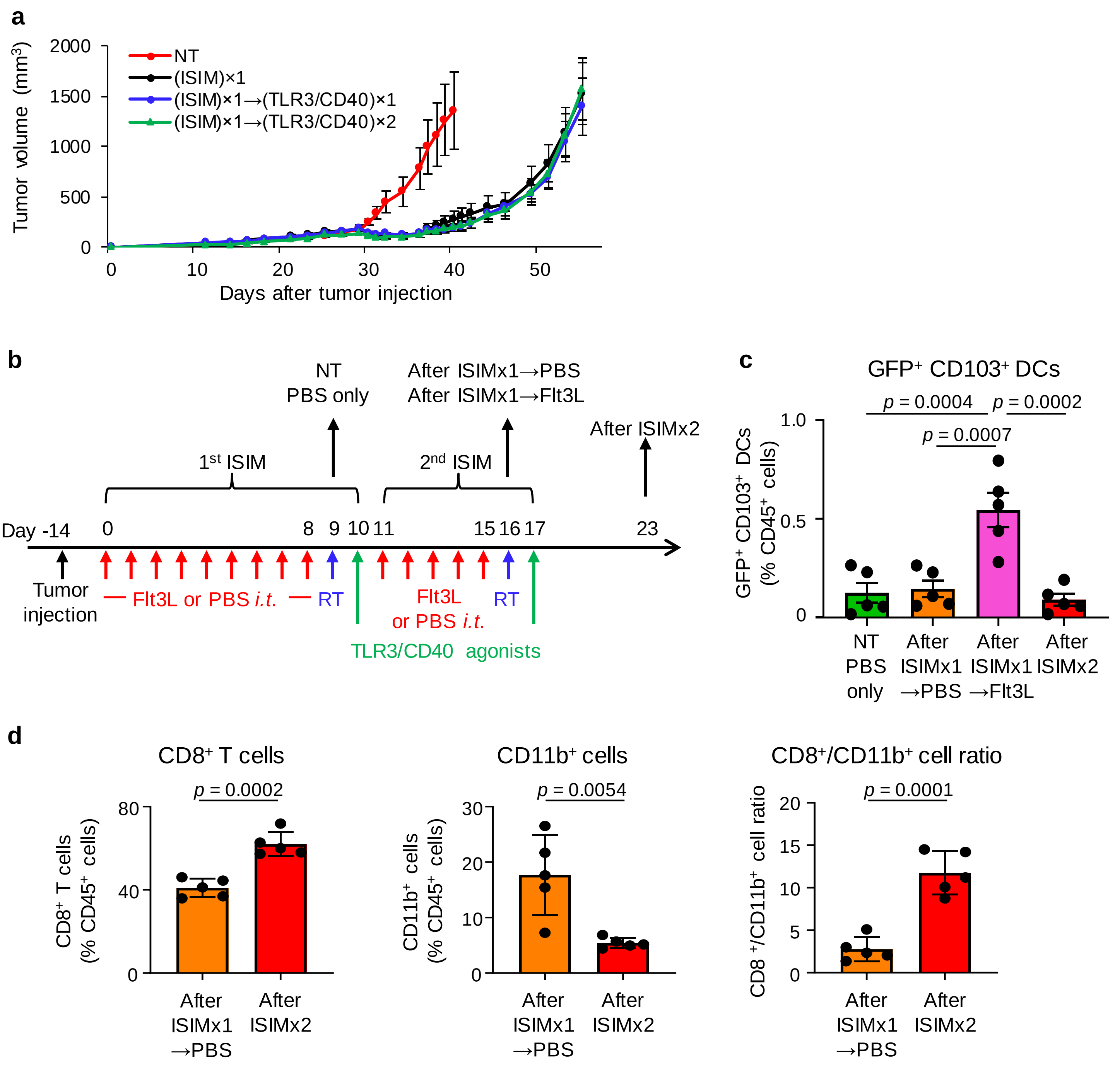

Supplementary Fig. 19 Serial *in situ* immunomodulation (ISIM) remodels tumor immune microenvironment. Related to Figure 10a.

a Tumor growth curves (mean) in AT-3 tumor-bearing mice in different treatment groups as indicated. n = 6 mice in all groups.

b Experimental set-up for serial ISIM. The timing of sample take down for analysis (**c, d**) was indicated.

c Frequency of GFP^+ $CD103^+$ DCs ($Ly6c^-$ class II^+ $CD11c^+$ $CD24^+$ $F4/80^-$ $CD103^+$ GFP^+) among $CD45^+$ cells in AT-3-GFP tumors treated with PBS intratumorally (*i.t.*) only for 9 days (NT PBS only), ISIM followed by PBS (After ISIMx1→PBS) or Flt3L *i.t.* injection (After ISIMx1→Flt3L) for 5 days, or ISIM twice (After ISIMx2). n = 5 mice in all groups.

d Frequency of $CD8^+$ T cells, $CD11b^+$ cells, and $CD8^+$ T cell/$CD11b^+$ cell ratio in AT-3-GFP tumors in different treatment groups as indicated. n = 5 mice in all groups. Statistical significance was determined by a one-way ANOVA with Tukey's multiple comparisons (**c**) and a two-tailed *t*-test (**d**). Mean ± SEM. Source data are provided as a Source Data file.

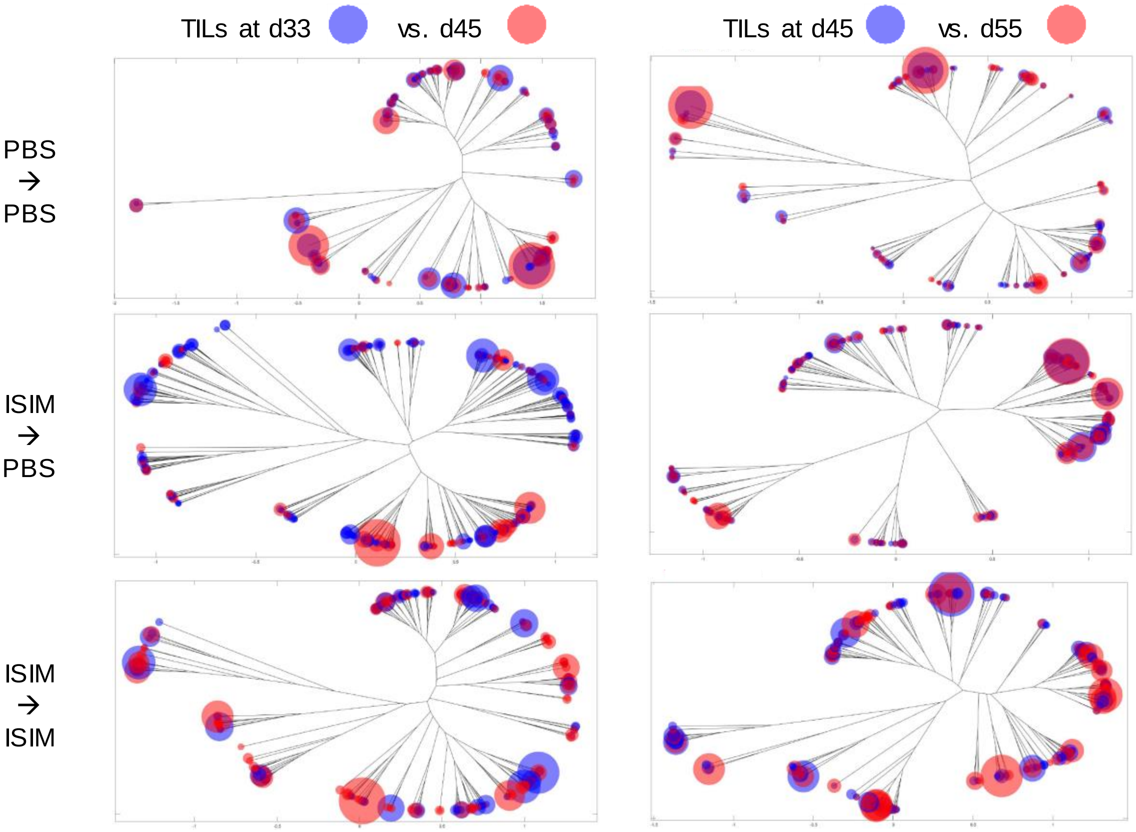

Supplementary Fig. 20 Serial *in situ* immunomodulation (ISIM) reshapes intratumoral TCR repertoires. Related to Figure 10b.

AT-3 tumor bearing mice were treated with PBS → PBS, ISIM → PBS, or ISIM → ISIM. Serial tumor biopsies were done before treatment at d33, after first treatment (PBS or ISIM) at d45, and after second treatment (PBS or ISIM) at d55. DNA from tumor tissue was extracted for TCRseq, and TCRseq data was analyzed using ImmunoMap[53]. The hierarchical clustering is visualized as overlapped weighted TCR repertoire dendrograms. Overlapped weighted TCR repertoire dendrograms visualize relatedness of sequences within repertoire along with relative frequency of CDR3 amino acid sequences. Each color represents AT-3 tumor biopsy from one individual animal at different time points: d33 (blue) and d45 (red) (left), and d45 (blue) and d55 (red) (right). The distance of the branch ends represents sequence distance, and the size of circles denotes frequency of sequence. Data shown are representative of three independent experiments.

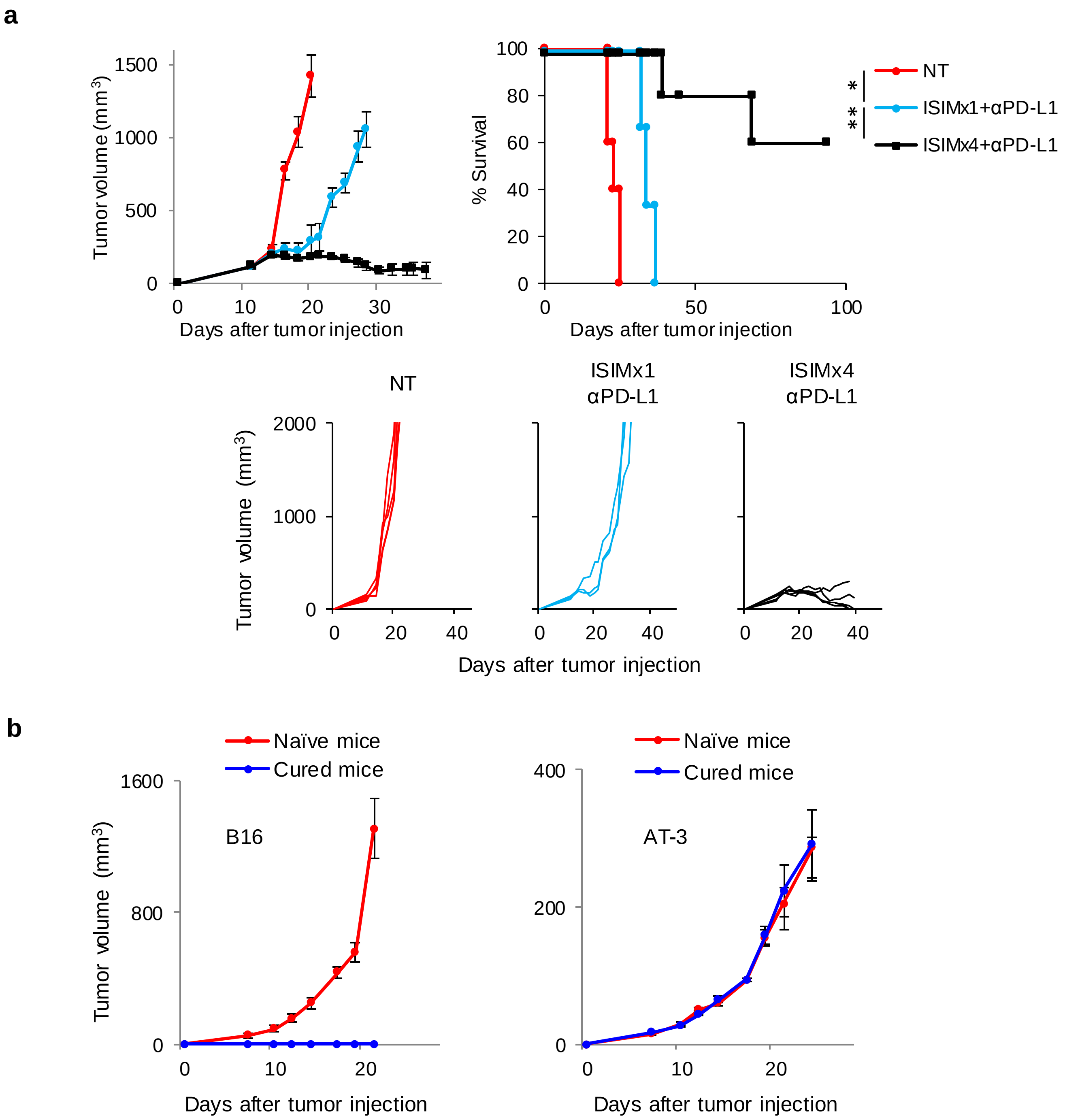

Supplementary Fig. 21 Serial *in situ* immunomodulation (ISIM) in combination with PD-L1 blockade eradicates established tumors and develops tumor-specific immunological memory in a B16 tumor model. Related to Figure 10c, d.

a, Tumor growth curves (mean and individual) and survival curves in B16 tumor-bearing mice in different treatment groups as indicated. n = 3 mice (ISIMx1 + αPD-L1) and 5 mice (any other groups).

b, Naïve C57BL/6 mice (n = 5) and surviving mice from (**a**) were rechallenged with B16 (left) and AT-3 (right) in the contralateral flank at d87 (B16) and on back at d94 (AT-3), respectively. *$p < 0.05$, **$p < 0.01$ by a log-rank test (**a**). Mean ± SEM. Source data are provided as a Source Data file.

Supplementary Table 1. The number of total templates, productive templates, total rearrangement, and productive rearrangement of each sample.
Only data from productive rearrangements were exported from the immunoSEQ Analyzer for further analysis.

Treatment	Mouse identification number	Timing of biopsy	Total templates	Productive templates	Total rearrangements	Productive rearrangements
PBS -> PBS	1	day33	32351	23090	12217	7846
PBS -> PBS	1	day45	8953	6038	3758	2342
PBS -> PBS	1	day55	9769	6443	3015	1884
PBS -> PBS	2	day33	2829	1793	1298	756
PBS -> PBS	2	day45	3523	2315	1734	1063
PBS -> PBS	2	day55	4289	2935	1873	1145
PBS -> PBS	3	day33	3169	2110	1105	607
PBS -> PBS	3	day45	18751	12119	8159	4892
PBS -> PBS	3	day55	2810	2022	1000	577
ISIM -> PBS	1	day33	5944	4362	1992	1175
ISIM -> PBS	1	day45	27792	20459	7545	4845
ISIM -> PBS	1	day55	13074	9153	4403	2760
ISIM -> PBS	2	day33	4135	2972	1354	833
ISIM -> PBS	2	day45	61194	43238	13411	8868
ISIM -> PBS	2	day55	22684	16439	6463	4090
ISIM -> PBS	3	day33	4518	2977	1944	1097
ISIM -> PBS	3	day45	27581	19346	8878	5464
ISIM -> PBS	3	day55	4051	2760	1634	924
ISIM -> ISIM	1	day33	4056	2834	1290	764
ISIM -> ISIM	1	day45	30882	22575	7954	5121
ISIM -> ISIM	1	day55	26048	18720	6163	4003
ISIM -> ISIM	2	day33	9141	6367	3893	2445
ISIM -> ISIM	2	day45	73674	54671	14856	9853
ISIM -> ISIM	2	day55	26383	18231	5665	3462
ISIM -> ISIM	3	day33	3911	2752	1466	842
ISIM -> ISIM	3	day45	7495	5416	2206	1250
ISIM -> ISIM	3	day55	4595	3358	1326	752
Average			16429.7	11685	4689.0	2950.4

Supplementary Table 2. Top five AT-3 tumor-infiltrating T-cell clones observed at different time points.

Top five most frequently detected CDR3β region sequences in AT-3 tumors at different time points are shown. Frequency is shown as productive frequency (%). Clones observed at multiple time points within the same individual mouse are highlighted by matching colors.

Treatment	Mouse identification number	CDR3β region sequence in AT-3 tumors on day 33	Frequency (%)	CDR3β region sequence in AT-3 tumors on day 45	Frequency (%)	CDR3β region sequence in AT-3 tumors on day 55	Frequency (%)
PBS -> PBS	1	CASSGNSGNTLYF	5.56	CASSGNSGNTLYF	8.41	CASSGNSGNTLYF	14.11
		CASRDRDQDTQYF	2.68	CGAGGVGSDYTF	6.36	CGAGGVGSDYTF	13.13
		CASGDGQYNNQAPLF	2.61	CASSERQFYEQYF	2.78	CASSLALYEQYF	2.76
		CASSLRPLGGGTGQLYF	2.60	CASSPGTGFEQYF	1.87	CASSPLGGPEQYF	1.80
		CGAGGVGSDYTF	2.26	CASSQGTNANSDYTF	1.47	CASSERQFYEQYF	1.66
PBS -> PBS	2	CASGARGGYEQYF	4.02	CASGARGGYEQYF	6.18	CASSGQGSNERLFF	4.77
		CSSSWDRGNERLFF	3.51	CASSGQGSNERLFF	3.11	CASSEDISYEQYF	4.36
		CASSSTGGYEQYF	2.51	CGAGGVGSDYTF	1.64	CASGARGGYEQYF	2.96
		CASSLPDANSDYTF	1.95	CASSLPDANSDYTF	1.60	CASSLPDANSDYTF	2.39
		CASSGQGSNERLFF	1.84	CASSGNSGNTLYF	1.56	CASSLAYEQYF	2.28
PBS -> PBS	3	CASKQLGGANTGQLYF	14.88	CASKQLGGANTGQLYF	9.14	CASKQLGGANTGQLYF	10.88
		CASSLGLVYEQYF	4.69	CASSQDTANERLFF	2.50	CASGDGQYNNQAPLF	8.75
		CASSPWGGDYAEQFF	4.21	CGVRWGSNERLFF	1.18	CASRDRDQDTQYF	7.81
		CASRDRDQDTQYF	4.12	CASSLGLVYEQYF	1.83	CASSFHRDYNSPLYF	4.99
		CASSFHRDYNSPLYF	2.51	CGASQGNYAEQFF	1.46	CASSPWGGDYAEQFF	3.06
ISIM -> PBS	1	CASSPWGGDYAEQFF	8.23	CASSLGDNNQAPLF	2.60	CAWSHRGANERLFF	2.79
		CASRDRDQDTQYF	6.21	CASRGGSNQAPLF	2.48	CAWSTGGASSYEQYF	1.65
		CASSPGRRGSDYTF	5.07	CASSFLGRQDTQYF	2.47	CASRGGSNQAPLF	1.27
		CASGDGQYNNQAPLF	4.45	CAWSHRGANERLFF	2.43	CASSLGDNNQAPLF	1.13
		CASSFHRDYNSPLYF	3.99	CASSPLGGPEQYF	2.37	CSSMTSYEQYF	1.09
ISIM -> PBS	2	CASSPGGAEQYF	4.07	CASSQDWGAYAEQFF	8.00	CASSQDWGAYAEQFF	8.85
		CASSLDTNSGNTLYF	4.07	CASSLGDRVGEQYF	3.56	CASSQGQISSYEQYF	4.18
		CASSFWQGGAETLYF	3.77	CASSQGQISSYEQYF	2.43	CASSDGYEQYF	2.82
		CASSGGLGGEQYF	1.85	CASSPWTSYEQYF	1.92	CASSLGDRVGEQYF	2.46
		CSSSLDRGHERLFF	1.85	CASREQNSGNTLYF	1.66	CASSQGLGEEQYF	2.04
ISIM -> PBS	3	CASSFHRDYNSPLYF	2.31	CASSTNWGNYAEQFF	3.80	CASSTNWGNYAEQFF	3.62
		CASSPDWGGSSQNTLYF	1.98	CASSLGRRSGNTLYF	2.16	CASSLGRRSGNTLYF	1.41
		CASRDRDQDTQYF	1.71	CASSSGEGAEQFF	1.31	CASSFHRDYNSPLYF	1.23
		CASGDGQYNNQAPLF	1.47	CASSEGTGRSSYEQYF	1.04	CASRDRDQDTQYF	1.12
		CASSPWGGDYAEQFF	1.31	CASGPPRGEQYF	1.00	CASSIRGDTQYF	1.12
ISIM -> ISIM	1	CASRDRDQDTQYF	6.74	CASSDAGNSYEQYF	2.42	CASSSDRANSDYTF	4.06
		CASGDGQYNNQAPLF	5.22	CASPTGTTYEQYF	1.90	CASSSLGGRGEQYF	2.41
		CASSFHRDYNSPLYF	3.63	CASSYTGGEQYF	1.79	CASSLGGDEQYF	2.15
		CASPTGTTYEQYF	2.43	CASGVSGLGGRYAEQFF	1.59	CASGDRRPYNNQAPLF	1.50
		CASSPWGGDYAEQFF	2.29	CTCSAGRGPSYEQYF	1.45	CASSIDRGGYEQYF	1.49
ISIM -> ISIM	2	CASSPGLVNTLYF	4.40	CASSLGLSNERLFF	5.54	CTCSAALGDYEQYF	4.66
		CASSKYEQYF	2.83	CASSFYRRGQAPLF	2.61	CASSLGLSNERLFF	4.06
		CASRDRDQDTQYF	2.78	CTCSVRGRNSPLYF	2.38	CASSLVGYEQYF	3.06
		CASGDGQYNNQAPLF	2.01	CASSDARGRDAEQFF	1.79	CASSYPDWGGWCDEQYF	2.59
		CASGRLDAEQFF	1.92	CASSGGGEQYF	1.65	CASSLDRANSDYTF	2.33
ISIM -> ISIM	3	CASSQEPFANSDYTF	9.30	CASRWGGDEQYF	12.00	CASSLDRANSDYTF	10.82
		CASRDRDQDTQYF	3.34	CASSLSYEQYF	4.32	CASSLPGASYEQYF	10.57
		CASSQETGNYAEQFF	2.97	CASRDRDQDTQYF	3.84	CASRWGGDEQYF	5.68
		CASSFHRDYNSPLYF	2.39	CASSPTGGANEQYF	3.30	CASSLSYEQYF	4.94
		CASSSSSYEQYF	1.88	CASSIGNTEVFF	2.71	CASSQEPFANSDYTF	2.91

Supplementary Table 3. List of antibodies

Antibodies	Source	Catlog Number	Dilution
Anti-mouse I-Ab clone AF6-120.1 FITC	BD	Cat# 553551	1:200
Anti-mouse CD44 clone IM7 FITC	Biolegend	Cat# 103002	1:200
Anti-mouse 4-1BB clone 17B5 PE	Biolegend	Cat# 106106	1:200
Anti-mouse CD8 alpha clone 53-6.7 BUV395	BD	Cat# 563786	1:400
Anti-mouse CD62L clone MEL-14 BV421	Biolegend	Cat# 104436	1:200
Anti-mouse CD3 clone 145-2C11 PerCP-Cy5.5	Biolegend	Cat# 100328	1:200
Anti-mouse CD45 clone 30-F11 Pacific Orange	Thermo Fisher scientific	Cat# MCD4530	1:200
Anti-mouse CD27 clone LG.3A10 PE-Cy7	Biolegend	Cat# 124216	1:200
Anti-mouse CX3CR1 clone SA011F11 APC	Biolegend	Cat# 149008	1:200
Anti-mouse PD-1 clone 29F.1A12 BV711	Biolegend	Cat# 135231	1:200
Anti-mouse CD4 clone GK1.5 BUV737	BD	Cat# 564298	1:400
Anti-mouse PD-L1 clone MIH5 PE	BD	Cat# 558091	1:200
Anti-mouse CD11c clone HL3 PE-Cy7	BD	Cat# 558079	1:200
Anti-mouse CD24 clone M1/69 BUV 737	BD	Cat# 565308	1:200
Anti-mouse F4/80 clone BM8 BV650	Biolegend	Cat# 123149	1:200
Anti-mouse F4/80 clone BM8 BV711	Biolegend	Cat# 123147	1:200
Anti-mouse Ly6G clone 1A8 BV711	BD	Cat# 563979	1:200
Anti-mouse CD4 clone GK1.5 FITC	Biolegend	Cat# 100406	1:400
Anti-mouse 4-1BB clone 17B5 Biotin	Biolegend	Cat# 106104	1:200
Anti-mouse CD86 clone GL-1 Alexa Fluor 488	Biolegend	Cat# 105108	1:200
Anti-mouse CD40 clone 3/23 BV421	BD	Cat# 562846	1:200
Anti-mouse CD103 clone 2E7 APC	Biolegend	Cat# 121414	1:200
Anti-mouse I-A/I-E clone M5/114.15.2 PerCP-Cy5.5	Biolegend	Cat# 107626	1:200
Anti-mouse I-Ad clone 39-10-8 FITC	Biolegend	Cat# 115005	1:200
Anti-mouse Ly6c clone HK1.4 BV711	Biolegend	Cat# 128037	1:200
Anti-mouse Thy1.2 clone 53-2.1 FITC	BD	Cat# 553003	1:400
Anti-mouse CD8 clone KT15 FITC	Thermo Fisher scientific	Cat# MA5-16759	1:400
Anti-mouse CD8 beta clone YTS156.7.7 APC-Cy7	Biolegend	Cat# 126620	1:400
Anti-mouse CD8 beta clone YTS156.7.7 FITC	Biolegend	Cat# 126606	1:400
Anti-mouse CD11b clone M1/70 BUV395	BD	Cat# 563553	1:200
Anti-mouse CD103 clone 2E7 PE	Biolegend	Cat# 121406	1:200
Anti-mouse CD8 alpha clone 53-6.7 BV510	Biolegend	Cat# 100752	1:400
Anti-mouse CD11b clone M1/70 PerCP-Cy5.5	BD	Cat# 550993	1:200
Anti-mouse Thy1.2 clone 53-2.1 PerCP-Cy5.5	Biolegend	Cat# 140322	1:400
Anti-mouse IFNγ clone XMG1.2 PerCP-Cy5.5	Thermo Fisher scientific	Cat# 45-7311-82	1:200
Anti-mouse TNFα clone MP6-XT22 PE-Cy7	Thermo Fisher scientific	Cat# 25-7321-80	1:200
Anti-mouse Tcf1 clone S33-966 PE	BD	Cat# 564217	1:200
LIVE/DEAD™ Fixable Near-IR Dead Cell Stain Kit	Thermo Fisher scientific	L34975	1:200
H-2Ld tetramer to peptide SPSYVYHQF MuLV env gp70 423-431 PE	NIH	N/A	1:50
