# Supplementary material for: Overcoming primary and acquired resistance to anti-PD-L1 therapy by induction and activation of tumor-residing cDC1s
Source: Nat Commun. 2020 Oct 27;11:5415. doi: 10.1038/s41467-020-19192-z (PMC7592056; doi:10.1038/s41467-020-19192-z)
Supplement: Supplementary file 2 — Description of Additional Supplementary Files [file 41467_2020_19192_MOESM2_ESM.pdf]

Description of Additional Supplementary Files

File Name: Supplementary Data 1. Gene expression patterns significantly associated with each cluster identified within the tumor-infiltrating immune cells ($CD45^+$ cells) in AT-3 tumor was determined by comparing cells from each respective cluster against all other cells. Differential expression was implemented in Seurat via the MAST method implemented in the FindMarkers function. Each row represents a single gene, and columns provide the raw p-value (p_val) of observed differences, the average log fold change (avg_logFC), the fraction of cells with positive detection of the given gene in the respective cluster (pct.1) or all other cells (pct.2), bonferroni adjusted P value (p_val_adj), and cluster number associated with the given gene (cluster).

File Name: Supplementary Data 2. Gene expression patterns significantly associated with Cluster 15 relative to cluster 16 were determined by comparing cells within each cluster. Differential expression was implemented in Seurat via the MAST method implemented in the FindMarkers function. Each row represents a single gene, and columns provide the raw p-value (p_val) of observed differences, the average log fold change (avg_logFC), the fraction of cells with positive detection of the given gene in the respective cluster (pct.1) or all other cells (pct.2), bonferroni adjusted P value (p_val_adj), and cluster number associated with the given gene (cluster).

File Name: Supplementary Data 3. Gene expression patterns significantly associated with each cluster identified within the lymphoid population subset (annotated lymphocyte cluster cells expressing *Cd3e* or *Ncr1*) of AT-3 tumor-infiltrating cells were determined by comparing cells

from each respective cluster against all other cells. Differential expression was implemented in Seurat via the MAST method implemented in the FindMarkers function. Each row represents a single gene, and columns provide the raw p-value (p_val) ofobserved differences, the average log fold change (avg_logFC), the fraction of cells with positive detection of the given gene in the respective cluster (pct.1) or all other cells (pct.2), bonferroni adjusted P value (p_val_adj), and cluster number associated with the given gene (cluster).

File Name: Supplementary Data 4. Gene expression patterns significantly associated with ISIM + anti-PD-L1 Ab-treatment relative to ISIM-treatment alone within Ly_C0 were determined by comparing cells within each cluster across the two treatment groups. Differential expression was implemented in Seurat via the MAST method implemented in the FindMarkers function. Each row represents a single gene, and columns provide the raw p-value (p_val) of observed differences, the average log fold change (avg_logFC), the fraction of cells with positive detection of the given gene in the respective cluster (pct.1) or all other cells (pct.2), and bonferroni adjusted P value (p_val_adj).

File Name: Supplementary Data 5. Gene set enrichment analysis (Using GO-Biological Processes, KEGG, REACTOME, BIOCARTA, PID, and HALLMARK pathways as reference) was performed on differential gene expression patterns associated with ISIM relative to NT (I vs NT) and ISIM + anti-PD-L1 Ab-treatment relative to ISIM-treatment alone (IP vs I) within each cluster identified from the lymphoid population subset (Ly_C0, 2, 4, 5, 6, 7, 10, 11). Each row represents a single pathway, and columns provide pathway name, ID, and description, set size, enrichment score, normalized enrichment score (NES), the raw p-value of observed differences,

adjusted p-value, q-value, rank, leading_edge, and core_enrichment. Each sub-table corresponds to the treatment related differential pathway identified in each respective cluster.
