# Supplementary material for: Overcoming primary and acquired resistance to anti-PD-L1 therapy by induction and activation of tumor-residing cDC1s
Source: Nat Commun. 2020 Oct 27;11:5415. doi: 10.1038/s41467-020-19192-z (PMC7592056; doi:10.1038/s41467-020-19192-z)
Supplement: Supplementary file 8 — Reporting Summary [file 41467_2020_19192_MOESM8_ESM.pdf]

nature research

Corresponding author(s): Fumito Ito, MD, PhD, FACS

Last updated by author(s): Sep 25, 2020

Reporting Summary

Nature Research wishes to improve the reproducibility of the work that we publish. This form provides structure for consistency and transparency in reporting. For further information on Nature Research policies, see Authors & Referees and the Editorial Policy Checklist .

Statistics

For all statistical analyses, confirm that the following items are present in the figure legend, table legend, main text, or Methods section.

n/a	Confirmed	
☐	☒	The exact sample size (*n*) for each experimental group/condition, given as a discrete number and unit of measurement
☐	☒	A statement on whether measurements were taken from distinct samples or whether the same sample was measured repeatedly
☐	☒	The statistical test(s) used AND whether they are one- or two-sided<br>*Only common tests should be described solely by name; describe more complex techniques in the Methods section.*
☒	☐	A description of all covariates tested
☐	☒	A description of any assumptions or corrections, such as tests of normality and adjustment for multiple comparisons
☐	☒	A full description of the statistical parameters including central tendency (e.g. means) or other basic estimates (e.g. regression coefficient) AND variation (e.g. standard deviation) or associated estimates of uncertainty (e.g. confidence intervals)
☐	☒	For null hypothesis testing, the test statistic (e.g. *F*, *t*, *r*) with confidence intervals, effect sizes, degrees of freedom and *P* value noted<br>*Give P values as exact values whenever suitable.*
☒	☐	For Bayesian analysis, information on the choice of priors and Markov chain Monte Carlo settings
☒	☐	For hierarchical and complex designs, identification of the appropriate level for tests and full reporting of outcomes
☒	☐	Estimates of effect sizes (e.g. Cohen's *d*, Pearson's *r*), indicating how they were calculated

Our web collection on statistics for biologists contains articles on many of the points above.

Software and code

Policy information about availability of computer code

Data collection	Zeiss Axio Imager Z1 (microscopy), FACS Diva 6 (flow cytometry), IVIS Spectrum imager (in vivo imaging), Illumina HiSeq2500 (scRNA seq), INSPIRE (200.1.681) (imaging flow cytometry)
Data analysis	Flow cytometric data analysis was performed using FlowJo version 10.4.2. TCRβ-Seq data analysis was performed using ImmunoSEQ Analyzer (v.3.0), ImmunoMap, LymphoSeq (v.1.14.0), vegan (v.2.5.6). ScRNA-seq data analysis was performed using Cell Ranger Single-Cell Software Suite (1.2), Seurat (3.1.5), scrublet (v.0.2.1), SingleR (v1.0.6), scater (v.1.14.6), AUCell (v.1.8.0), clusterProfiler (v.3.14.3), IDEAS software (6.2) for imaging flow cytometry, Living Image (4.3.1) for IVIS Spectrum imager. Statistical software were run using GraphPad prizm 8.0.2, R version 3.6.1 or MATLAB R2019a.

For manuscripts utilizing custom algorithms or software that are central to the research but not yet described in published literature, software must be made available to editors/reviewers. We strongly encourage code deposition in a community repository (e.g. GitHub). See the Nature Research guidelines for submitting code & software for further information.

Data

Policy information about availability of data

All manuscripts must include a data availability statement. This statement should provide the following information, where applicable:

- Accession codes, unique identifiers, or web links for publicly available datasets
- A list of figures that have associated raw data
- A description of any restrictions on data availability

Raw scRNAseq data supporting the findings of this study have been deposited in the National Center for Biotechnology Information Gene Expression Omnibus (NCBI-GEO) under accession number GSE154879 at https://www.ncbi.nlm.nih.gov/geo/query/acc.cgi?acc=GSE154879. The TCRseq data are available at https://github.com/mdlong-rpccc/Ito_ISIM. The source data underlying graphs in Figs. 1–10 and Supplementary Figs. 1, 3-10, 18, 19 and 21 has been provided as a source data file. All data generated and analyzed are available from the corresponding author upon reasonable request. Databases used for collecting gene and/or

functional pathway information include the Mouse Genome Informatics database (http://www.informatics.jax.org/) and the Molecular Signatures Database (https://www.gsea-msigdb.org/gsea/msigdb).

Field-specific reporting

Please select the one below that is the best fit for your research. If you are not sure, read the appropriate sections before making your selection.

☒ Life sciences ☐ Behavioural & social sciences ☐ Ecological, evolutionary & environmental sciences

For a reference copy of the document with all sections, see nature.com/documents/nr-reporting-summary-flat.pdf

Life sciences study design

All studies must disclose on these points even when the disclosure is negative.

Sample size	Sample sizes were determined based on our and other investigators experience with the respective cell lines used (e.g.: Saito et al. 2016 (PMID: 27197199), Yamauchi et al. 2020 (PMID: 32255766), Oba et al. 2020 (PMID: 32848036), Mohammadpour et al. 2019 (PMID: 31566578), Twum et al. 2019 (PMID: 30728331), Ngiow et al 2015 (PMID: 26208901), Juneja et al. 2017 (PMID: 28302645)). No statistical methods were used as we observed many statistically significant effects in the data with the above methods of sample size selection without a priori sample size calculations.
Data exclusions	Any mice bearing tumors <50 mm3 were excluded from the experiment.
Replication	All experiments were successfully reproduced. We used scRNAseq to confirm some of the findings obtained from flow cytometry analysis. We attempted to validate and reproduce new findings obtained from scRNAseq by flow cytometry analysis (e.g. Slamf6, Tcf1 expression in tumor-infiltrating lymphocytes)
Randomization	Stratified randomization was used based on tumor size to ensure equal distribution of tumor sizes within each group.
Blinding	TCRseq analyses were performed in a blinded fashion.

Reporting for specific materials, systems and methods

We require information from authors about some types of materials, experimental systems and methods used in many studies. Here, indicate whether each material, system or method listed is relevant to your study. If you are not sure if a list item applies to your research, read the appropriate section before selecting a response.

Materials & experimental systems

n/a	Involved in the study	
☐	☒	Antibodies
☐	☒	Eukaryotic cell lines
☒	☐	Palaeontology
☐	☒	Animals and other organisms
☒	☐	Human research participants
☒	☐	Clinical data

Methods

n/a	Involved in the study	
☒	☐	ChIP-seq
☐	☒	Flow cytometry
☒	☐	MRI-based neuroimaging

Antibodies

Antibodies used

anti-mouse:

flow cytometry: all surface antibodies were used at 1:200, except for CD4, CD8, and Thy1.2 (1:400); all intracellular antibodies were used at 1:200.

Biolegend: CD44 clone (IM7 FITC, # 103022), 4-1BB (clone 17B5 PE, # 106106), CD62L (clone MEL-14 BV421, # 104436), CD3 (clone 145-2C11 PerCP-Cy5.5, # 100328), CX3CR1 (clone SA011F11 APC, # 149008), PD-1 (clone 29F.1A12 BV711, # 135231), F4/80 (clone BM8 BV650, # 123149), CD4 (clone GK1.5 FITC, # 100406), CD86 (clone GL-1 Alexa Fluor 488, # 105018), CD103 (clone 2E7 APC, # 121414), I-A/I-E (clone M5/114.15.2 PerCP-Cy5.5, # 107626), I-Ad (clone 39-10-8 FITC, # 115005), Ly6c (clone HK1.4 BV711, # 128037), CD8 beta (clone YTS156.7.7 APC-Cy7, # 126620), CD8 beta (clone YTS156.7.7 FITC, # 126606), CD103 (clone 2E7 PE, # 121406), CD8 alpha (clone 53-6.7 BV510, # 100752), Thy1.2 (clone 53-2.1 PerCP-Cy5.5, # 140322),

BD:I-Ab (clone AF6-120.1 FITC, # 553551), CD4 (clone GK1.5 BUV737, # 564298), CD8 alpha (clone 53-6.7 BUV395, # 563786), PD-L1 (clone MIH5 PE, # 558091), CD11c (clone HL3 PE-Cy7, # 558079), CD24 (clone M1/69 BUV 737, # 565308), Ly6G (clone 1A8 BV711, # 563979), CD40 (clone 3/23 BV421, # 562846), Thy1.2 (clone 53-2.1 FITC, # 553003), CD11b (clone M1/70 BUV395, # 563553),

Thermo Fisher scientific: CD45 (clone 30-F11 Pacific Orange, # MCD4530), CD8 (clone KT15 FITC, # MA5-16759), IFNγ (clone XMG1.2 PerCP-Cyanine5.5, # 45-7311-82), TNFα (clone MP6-XT22 PE-Cy7, # 25-7321-80), LIVE/DEADTM Fixable Near-IR Dead Cell Stain Kit (L34975)

depletion studies:
BioXCell: CD8 beta (clone 53-5.8, # BE0223), CD4 (clone GK1.5, # BE0003-1), NK1.1 (clone PK136, # BE0036), IL-12 p40 (clone C17.8, # BE0051), IFNγ (clone R4-6A2, # BE0054), isotype control (IgG2a, # BE0085), isotype control (IgG2b, # BE0090)

in vivo treatment:
BioXCell: PD-L1 (clone 10F.9G2, # BE0101), rat IgG2b isotype control (clone LTF-2, # BE0090), CD40 (clone FGK4.5, # BE0016-2)

immunohistochemistry:
Cell signaling: CD8 alpha (clone D4W2Z, # 98941S),
Abcam: CD163 (clone EPR19518, # ab182422)

Validation

all primary anti-mouse antibodies were validated for flow cytometry by the manufacturers.
CD44 clone (IM7 FITC, # 103022) https://www.biolegend.com/en-us/search-results/fitc-anti-mouse-human-cd44-antibody-314
4-1BB (clone 17B5 PE, # 106106) https://www.biolegend.com/en-us/products/pe-anti-mouse-cd137-antibody-51
CD62L (clone MEL-14 BV421, # 104436), https://www.biolegend.com/en-us/products/brilliant-violet-421-anti-mouse-cd62l-antibody-7164
CD3 (clone 145-2C11 PerCP-Cy5.5, # 100328), https://www.biolegend.com/en-us/products/percp-cyanine5-5-anti-mouse-cd3epsilon-antibody-4191
CX3CR1 (clone SA011F11 APC, # 149008), https://www.biolegend.com/en-us/products/apc-anti-mouse-cx3cr1-antibody-10460
PD-1 (clone 29F.1A12 BV711, # 135231), https://www.biolegend.com/en-us/products/brilliant-violet-711-anti-mouse-cd279-pd-1-antibody-12303
F4/80 (clone BM8 BV650, # 123149), https://www.biolegend.com/en-us/products/brilliant-violet-650-anti-mouse-f4-80-antibody-10630
CD4 (clone GK1.5 FITC, # 100406), https://www.biolegend.com/en-us/products/fitc-anti-mouse-cd4-antibody-248
CD86 (clone GL-1 Alexa Fluor 488, # 105018), https://www.biolegend.com/en-us/products/alexa-fluor-488-anti-mouse-cd86-antibody-3120
CD103 (clone 2E7 APC, # 121414), https://www.biolegend.com/en-us/products/apc-anti-mouse-cd103-antibody-4914
I-A/I-E (clone M5/114.15.2 PerCP-Cy5.5, # 107626), https://www.biolegend.com/en-us/products/percp-cyanine5-5-anti-mouse-i-a-i-e-antibody-4282
I-Ad (clone 39-10-8 FITC, # 115005), https://www.biolegend.com/en-us/search-results/fitc-anti-mouse-i-ad-antibody-1897
Ly6c (clone HK1.4 BV711, # 128037), https://www.biolegend.com/en-us/products/brilliant-violet-711-anti-mouse-ly-6c-antibody-8935
CD8 beta (clone YTS156.7.7 APC-Cy7, # 126620), https://www.biolegend.com/en-us/products/apc-cyanine7-anti-mouse-cd8b-antibody-10021
CD8 beta (clone YTS156.7.7 FITC, # 126606), https://www.biolegend.com/en-us/products/fitc-anti-mouse-cd8b-antibody-4475
CD103 (clone 2E7 PE, # 121406), https://www.biolegend.com/en-us/products/pe-anti-mouse-cd103-antibody-3574
CD8 alpha (clone 53-6.7 BV510, # 100752), https://www.biolegend.com/en-us/products/brilliant-violet-510-anti-mouse-cd8a-antibody-7992
Thy1.2 (clone 53-2.1 PerCP-Cy5.5, # 140322), https://www.biolegend.com/en-us/products/percp-cyanine5-5-anti-mouse-cd902-thy-12-antibody-8993

BD:
I-Ab (clone AF6-120.1 FITC, # 553551), https://www.bdbiosciences.com/us/reagents/research/antibodies-buffers/immunology-reagents/anti-mouse-antibodies/cell-surface-antigens/fitc-mouse-anti-mouse-i-ab-af6-1201/p/553551
CD4 (clone GK1.5 BUV737, # 564298), https://www.bdbiosciences.com/us/reagents/research/antibodies-buffers/immunology-reagents/anti-mouse-antibodies/cell-surface-antigens/buv737-rat-anti-mouse-cd4-gk15/p/612761
CD8 alpha (clone 53-6.7 BUV395, # 563786), https://www.bdbiosciences.com/us/reagents/research/antibodies-buffers/immunology-reagents/anti-mouse-antibodies/cell-surface-antigens/buv395-rat-anti-mouse-cd8a-53-67/p/563786
PD-L1 (clone MIH5 PE, # 558091), https://www.bdbiosciences.com/us/applications/research/b-cell-research/surface-markers/mouse/pe-rat-anti-mouse-cd274-mih5/p/558091
CD11c (clone HL3 PE-Cy7, # 558079), https://www.bdbiosciences.com/us/reagents/research/antibodies-buffers/immunology-reagents/anti-mouse-antibodies/cell-surface-antigens/pe-cy7-hamster-anti-mouse-cd11c-hl3/p/558079
CD24 (clone M1/69 BUV 737, # 565308), https://www.bdbiosciences.com/us/reagents/research/antibodies-buffers/immunology-reagents/anti-mouse-antibodies/cell-surface-antigens/buv737-rat-anti-mouse-cd24-m169/p/612832
Ly6G (clone 1A8 BV711, # 563979), https://www.bdbiosciences.com/us/reagents/research/antibodies-buffers/immunology-reagents/anti-mouse-antibodies/cell-surface-antigens/bv711-rat-anti-mouse-ly-6g-1a8/p/563979
CD40 (clone 3/23 BV421, # 562846), https://www.bdbiosciences.com/us/applications/research/b-cell-research/surface-markers/mouse/bv421-rat-anti-mouse-cd40-323/p/562846
Thy1.2 (clone 53-2.1 FITC, # 553003), https://www.bdbiosciences.com/us/applications/research/stem-cell-research/cancer-research/mouse/fitc-rat-anti-mouse-cd902-53-21/p/553003
CD11b (clone M1/70 BUV395, # 563553),https://www.bdbiosciences.com/us/applications/research/stem-cell-research/mesenchymal-stem-cell-markers-bone-marrow/mouse/negative-markers/buv395-rat-anti-cd11b-m170/p/563553

Thermo Fisher scientific:
CD45 (clone 30-F11 Pacific Orange, # MCD4530), https://www.thermofisher.com/antibody/product/CD45-Antibody-clone-30-F11-Monoclonal/MCD4530
CD8 (clone KT15 FITC, # MA5-16759), https://www.thermofisher.com/antibody/product/CD8-alpha-Antibody-clone-KT15-

Monoclonal/MA5-16759
IFNγ (clone XMG1.2 PerCP-Cyanine5.5, # 45-7311-82), https://www.thermofisher.com/antibody/product/IFN-gamma-Antibody-clone-XMG1-2-Monoclonal/45-7311-82
TNFα (clone MP6-XT22 PE-Cy7, # 25-7321-80), https://www.thermofisher.com/antibody/product/TNF-alpha-Antibody-clone-MP6-XT22-Monoclonal/25-7321-80
LIVE/DEAD Fixable Near-IR Dead Cell Stain Kit (L34975) https://www.thermofisher.com/order/catalog/product/L34976#/L34976

depletion studies:
BioXCell:
CD8 beta (clone 53-5.8, # BE0223), https://bxcell.com/product/m-lyt-3-2-ly-3-2/
CD4 (clone GK1.5, # BE0003-1), https://bxcell.com/product/m-cd4/
NK1.1 (clone PK136, # BE0036), https://bxcell.com/product/nk-1-1/
IL-12 p40 (clone C17.8, # BE0051), https://bxcell.com/product/invivomab-anti-m-il-12-il-23/
IFNγ (clone R4-6A2, # BE0054), https://bxcell.com/product/m-inf-gamma/
isotype control (IgG2a, # BE0085), https://bxcell.com/product/invivomab-mouse-igg2a-isotype-control-unknown-specificity/
isotype control (IgG2b, # BE0090), https://bxcell.com/product/rat-igg2b-isotype-control/

in vivo treatment:
BioXCell: PD-L1 (clone 10F.9G2, # BE0101), https://bxcell.com/product/m-pdl-1/
rat IgG2b isotype control (clone LTF-2, # BE0090), https://bxcell.com/product/rat-igg2b-isotype-control/
CD40 (clone FGK4.5, # BE0016-2) https://bxcell.com/product/m-cd40/

immunohistochemistry:
Cell signaling: CD8 alpha (clone D4W2Z, # 98941S), https://www.cellsignal.com/products/primary-antibodies/cd8a-d4w2z-xp-rabbit-mab-mouse-specific/98941?Ntk=Products&Ntt=98941
Abcam: CD163 (clone EPR19518, # ab182422) https://www.abcam.com/cd163-antibody-epr19518-ab182422.html

Eukaryotic cell lines

Policy information about cell lines

Cell line source(s)	4T1 and B16-F10 tumor cell lines were purchased from the American Type Culture Collection (ATCC). AT-3 tumor cell line was established from a primary mammary gland carcinoma of MTAG (MMTV-PyMT/B6) mice (PMID: 17709499). MC38 colon adenocarcinoma cell line was gift from Weiping Zou (University of Michigan). 4T1-luc was generated in-house using lentiviral transduction.<br>AT-3-GFP was generated in-house using retroviral transduction.
Authentication	Cell lines obtained from external institutions were authenticated by morphology, phenotype and growth.
Mycoplasma contamination	Cells lines tested negative for mycoplasma contamination prior to sample generation. Samples were confirmed negative using MycoAlert (Lonza) Mycoplasma Detection Kit.
Commonly misidentified lines (See ICLAC register)	No commonly misidentified cell lines were used.

Animals and other organisms

Policy information about studies involving animals; ARRIVE guidelines recommended for reporting animal research

Laboratory animals	C57BL/6J, Pmel-1 TCR-transgenic mice (B6.Cg Thy1a-Tg(TcraTcrb)8Rest/J), Balb/c, C57BL/6J-Batf3-/-, all female, all 7-10 weeks old at the beginning of each experiment, and were housed in the Unit for Laboratory Animal Medicine at the Roswell Park Comprehensive Cancer Center in compliance with the Institutional Animal Care and Use Committee regulations. Housing conditions at the Roswell Park Comprehensive Cancer Center: Mice were maintained in a specific pathogen free unit on a 12hr light: 12hr dark cycle. The animal rooms are provided with 100% fresh, HEPA filtered air at 10-15 air changes per hour. Room temperatures are controlled by reheat units within each room, and are maintained within the range of 70°F ± 2° F. The Humidity levels are controlled globally, and it is maintained between 30-70%.
Wild animals	No wild animals were used in this study.
Field-collected samples	No field-collected samples were used in this study.
Ethics oversight	All experiments were reviewed and approved by the Institutional Animal Care and Use Committee (IACUC) of the Roswell Park Comprehensive Cancer Center.

Note that full information on the approval of the study protocol must also be provided in the manuscript.

Flow Cytometry

Plots

Confirm that:

- [x] The axis labels state the marker and fluorochrome used (e.g. CD4-FITC).
- [x] The axis scales are clearly visible. Include numbers along axes only for bottom left plot of group (a 'group' is an analysis of identical markers).
- [x] All plots are contour plots with outliers or pseudocolor plots.
- [x] A numerical value for number of cells or percentage (with statistics) is provided.

Methodology

Sample preparation	Mouse blood, lymph nodes (LN), and tumors were harvested 5-7 days after in situ immunomodulation (ISIM) unless specified otherwise. Single cell suspensions filtered by 40µm nylon mesh were prepared for flow cytometric analysis. Red blood cells in blood were lysed using ACK Lysis Buffer (Life Technologies).
Instrument	BD LSR Fortessa
Software	collection: FACS DIva 6<br>analysis: Flow Jo 10.4.2
Cell population abundance	Dead cells and doublets were excluded on the basis of forward and side scatter and Fixable Live/Dead NearIR. Purity of sorted cells for scRNAseq was >95%
Gating strategy	Cells were first gated in intact cells using FSC/SCC, doublets were excluded using FSC-W vs SSC-A, cells were then gated on live cells using LIVE/DEAD Fixable Near-IR Dead Cell Stain Kit , followed by cell type specific gating using fluorescently labeled antibodies.

- [x] Tick this box to confirm that a figure exemplifying the gating strategy is provided in the Supplementary Information.
